# Association of biological sex with clinical outcomes and biomarkers of Alzheimer’s disease in adults with Down syndrome

**DOI:** 10.1093/braincomms/fcad074

**Published:** 2023-03-17

**Authors:** M Florencia Iulita, Alexandre Bejanin, Eduard Vilaplana, Maria Carmona-Iragui, Bessy Benejam, Laura Videla, Isabel Barroeta, Susana Fernández, Miren Altuna, Jordi Pegueroles, Victor Montal, Silvia Valldeneu, Sandra Giménez, Sofía González-Ortiz, Soraya Torres, Shaimaa El Bounasri El Bennadi, Concepcion Padilla, Mateus Rozalem Aranha, Teresa Estellés, Ignacio Illán-Gala, Olivia Belbin, Natalia Valle-Tamayo, Valle Camacho, Esther Blessing, Ricardo S Osorio, Sebastian Videla, Sylvain Lehmann, Anthony J Holland, Henrik Zetterberg, Kaj Blennow, Daniel Alcolea, Jordi Clarimón, Shahid H Zaman, Rafael Blesa, Alberto Lleó, Juan Fortea

**Affiliations:** Sant Pau Memory Unit, Department of Neurology, Hospital de la Santa Creu i Sant Pau, Biomedical Research Institute Sant Pau, Universitat Autònoma de Barcelona, Barcelona 08025, Spain; Center of Biomedical Investigation Network for Neurodegenerative Diseases (CIBERNED), Madrid 28031, Spain; Women’s Brain Project, Guntershausen 8357, Switzerland; Sant Pau Memory Unit, Department of Neurology, Hospital de la Santa Creu i Sant Pau, Biomedical Research Institute Sant Pau, Universitat Autònoma de Barcelona, Barcelona 08025, Spain; Center of Biomedical Investigation Network for Neurodegenerative Diseases (CIBERNED), Madrid 28031, Spain; Sant Pau Memory Unit, Department of Neurology, Hospital de la Santa Creu i Sant Pau, Biomedical Research Institute Sant Pau, Universitat Autònoma de Barcelona, Barcelona 08025, Spain; Center of Biomedical Investigation Network for Neurodegenerative Diseases (CIBERNED), Madrid 28031, Spain; Sant Pau Memory Unit, Department of Neurology, Hospital de la Santa Creu i Sant Pau, Biomedical Research Institute Sant Pau, Universitat Autònoma de Barcelona, Barcelona 08025, Spain; Center of Biomedical Investigation Network for Neurodegenerative Diseases (CIBERNED), Madrid 28031, Spain; Barcelona Down Medical Center, Fundació Catalana Síndrome de Down, Barcelona 08029, Spain; Center of Biomedical Investigation Network for Neurodegenerative Diseases (CIBERNED), Madrid 28031, Spain; Barcelona Down Medical Center, Fundació Catalana Síndrome de Down, Barcelona 08029, Spain; Sant Pau Memory Unit, Department of Neurology, Hospital de la Santa Creu i Sant Pau, Biomedical Research Institute Sant Pau, Universitat Autònoma de Barcelona, Barcelona 08025, Spain; Center of Biomedical Investigation Network for Neurodegenerative Diseases (CIBERNED), Madrid 28031, Spain; Barcelona Down Medical Center, Fundació Catalana Síndrome de Down, Barcelona 08029, Spain; Sant Pau Memory Unit, Department of Neurology, Hospital de la Santa Creu i Sant Pau, Biomedical Research Institute Sant Pau, Universitat Autònoma de Barcelona, Barcelona 08025, Spain; Center of Biomedical Investigation Network for Neurodegenerative Diseases (CIBERNED), Madrid 28031, Spain; Barcelona Down Medical Center, Fundació Catalana Síndrome de Down, Barcelona 08029, Spain; Sant Pau Memory Unit, Department of Neurology, Hospital de la Santa Creu i Sant Pau, Biomedical Research Institute Sant Pau, Universitat Autònoma de Barcelona, Barcelona 08025, Spain; Center of Biomedical Investigation Network for Neurodegenerative Diseases (CIBERNED), Madrid 28031, Spain; Sant Pau Memory Unit, Department of Neurology, Hospital de la Santa Creu i Sant Pau, Biomedical Research Institute Sant Pau, Universitat Autònoma de Barcelona, Barcelona 08025, Spain; Center of Biomedical Investigation Network for Neurodegenerative Diseases (CIBERNED), Madrid 28031, Spain; Sant Pau Memory Unit, Department of Neurology, Hospital de la Santa Creu i Sant Pau, Biomedical Research Institute Sant Pau, Universitat Autònoma de Barcelona, Barcelona 08025, Spain; Center of Biomedical Investigation Network for Neurodegenerative Diseases (CIBERNED), Madrid 28031, Spain; Sant Pau Memory Unit, Department of Neurology, Hospital de la Santa Creu i Sant Pau, Biomedical Research Institute Sant Pau, Universitat Autònoma de Barcelona, Barcelona 08025, Spain; Center of Biomedical Investigation Network for Neurodegenerative Diseases (CIBERNED), Madrid 28031, Spain; Multidisciplinary Sleep Unit, Hospital de la Santa Creu i Sant Pau, Barcelona 08041, Spain; Hospital del Mar, Barcelona 08003, Spain; Sant Pau Memory Unit, Department of Neurology, Hospital de la Santa Creu i Sant Pau, Biomedical Research Institute Sant Pau, Universitat Autònoma de Barcelona, Barcelona 08025, Spain; Center of Biomedical Investigation Network for Neurodegenerative Diseases (CIBERNED), Madrid 28031, Spain; Sant Pau Memory Unit, Department of Neurology, Hospital de la Santa Creu i Sant Pau, Biomedical Research Institute Sant Pau, Universitat Autònoma de Barcelona, Barcelona 08025, Spain; Center of Biomedical Investigation Network for Neurodegenerative Diseases (CIBERNED), Madrid 28031, Spain; Sant Pau Memory Unit, Department of Neurology, Hospital de la Santa Creu i Sant Pau, Biomedical Research Institute Sant Pau, Universitat Autònoma de Barcelona, Barcelona 08025, Spain; Center of Biomedical Investigation Network for Neurodegenerative Diseases (CIBERNED), Madrid 28031, Spain; Sant Pau Memory Unit, Department of Neurology, Hospital de la Santa Creu i Sant Pau, Biomedical Research Institute Sant Pau, Universitat Autònoma de Barcelona, Barcelona 08025, Spain; Center of Biomedical Investigation Network for Neurodegenerative Diseases (CIBERNED), Madrid 28031, Spain; Sant Pau Memory Unit, Department of Neurology, Hospital de la Santa Creu i Sant Pau, Biomedical Research Institute Sant Pau, Universitat Autònoma de Barcelona, Barcelona 08025, Spain; Center of Biomedical Investigation Network for Neurodegenerative Diseases (CIBERNED), Madrid 28031, Spain; Sant Pau Memory Unit, Department of Neurology, Hospital de la Santa Creu i Sant Pau, Biomedical Research Institute Sant Pau, Universitat Autònoma de Barcelona, Barcelona 08025, Spain; Center of Biomedical Investigation Network for Neurodegenerative Diseases (CIBERNED), Madrid 28031, Spain; Sant Pau Memory Unit, Department of Neurology, Hospital de la Santa Creu i Sant Pau, Biomedical Research Institute Sant Pau, Universitat Autònoma de Barcelona, Barcelona 08025, Spain; Center of Biomedical Investigation Network for Neurodegenerative Diseases (CIBERNED), Madrid 28031, Spain; Sant Pau Memory Unit, Department of Neurology, Hospital de la Santa Creu i Sant Pau, Biomedical Research Institute Sant Pau, Universitat Autònoma de Barcelona, Barcelona 08025, Spain; Center of Biomedical Investigation Network for Neurodegenerative Diseases (CIBERNED), Madrid 28031, Spain; Nuclear Medicine Department, Hospital de la Santa Creu i Sant Pau, Barcelona 08041, Spain; Department of Psychiatry, NYU Grossman School of Medicine, New York, NY 10016, USA; Department of Psychiatry, NYU Grossman School of Medicine, New York, NY 10016, USA; Clinical Research Support Unit, Bellvitge Biomedical Research Institute (IDIBELL), Department of Clinical Pharmacology, University of Barcelona, Barcelona 08908, Spain; Institute for Neurosciences of Montpellier, Institute for Regenerative Medicine and Biotherapy, Université de Montpellier, CHU de Montpellier, INSERM, Montpellier 34295, France; Department of Psychiatry, Cambridge Intellectual and Developmental Disabilities Research Group, University of Cambridge, Douglas House, Cambridge CB2 8AH, United Kingdom; Cambridgeshire & Peterborough NHS Foundation Trust, Fulbourn Hospital, Cambridge CB21 5EF, United Kingdom; Department of Psychiatry and Neurochemistry, University of Gothenburg, Möndal 40530, Sweden; Clinical Neurochemistry Laboratory, Sahlgrenska University Hospital, Mölndal 40530, Sweden; UK Dementia Research Institute, University College London, London WC1E 6BT, United Kingdom; Department of Neurodegenerative Disease, University College London Institute of Neurology, London WC1E 6BT, United Kingdom; Hong Kong Center for Neurodegenerative Diseases, Clear Water Bay, Hong Kong 1512-1518, China; Department of Psychiatry and Neurochemistry, University of Gothenburg, Möndal 40530, Sweden; Clinical Neurochemistry Laboratory, Sahlgrenska University Hospital, Mölndal 40530, Sweden; Sant Pau Memory Unit, Department of Neurology, Hospital de la Santa Creu i Sant Pau, Biomedical Research Institute Sant Pau, Universitat Autònoma de Barcelona, Barcelona 08025, Spain; Center of Biomedical Investigation Network for Neurodegenerative Diseases (CIBERNED), Madrid 28031, Spain; Sant Pau Memory Unit, Department of Neurology, Hospital de la Santa Creu i Sant Pau, Biomedical Research Institute Sant Pau, Universitat Autònoma de Barcelona, Barcelona 08025, Spain; Center of Biomedical Investigation Network for Neurodegenerative Diseases (CIBERNED), Madrid 28031, Spain; Department of Psychiatry, Cambridge Intellectual and Developmental Disabilities Research Group, University of Cambridge, Douglas House, Cambridge CB2 8AH, United Kingdom; Cambridgeshire & Peterborough NHS Foundation Trust, Fulbourn Hospital, Cambridge CB21 5EF, United Kingdom; Sant Pau Memory Unit, Department of Neurology, Hospital de la Santa Creu i Sant Pau, Biomedical Research Institute Sant Pau, Universitat Autònoma de Barcelona, Barcelona 08025, Spain; Center of Biomedical Investigation Network for Neurodegenerative Diseases (CIBERNED), Madrid 28031, Spain; Sant Pau Memory Unit, Department of Neurology, Hospital de la Santa Creu i Sant Pau, Biomedical Research Institute Sant Pau, Universitat Autònoma de Barcelona, Barcelona 08025, Spain; Center of Biomedical Investigation Network for Neurodegenerative Diseases (CIBERNED), Madrid 28031, Spain; Sant Pau Memory Unit, Department of Neurology, Hospital de la Santa Creu i Sant Pau, Biomedical Research Institute Sant Pau, Universitat Autònoma de Barcelona, Barcelona 08025, Spain; Center of Biomedical Investigation Network for Neurodegenerative Diseases (CIBERNED), Madrid 28031, Spain; Barcelona Down Medical Center, Fundació Catalana Síndrome de Down, Barcelona 08029, Spain

**Keywords:** Down syndrome, Alzheimer’s disease, sex, gender, precision medicine

## Abstract

The study of sex differences in Alzheimer’s disease is increasingly recognized as a key priority in research and clinical development. People with Down syndrome represent the largest population with a genetic link to Alzheimer’s disease (>90% in the 7th decade). Yet, sex differences in Alzheimer’s disease manifestations have not been fully investigated in these individuals, who are key candidates for preventive clinical trials. In this double-centre, cross-sectional study of 628 adults with Down syndrome [46% female, 44.4 (34.6; 50.7) years], we compared Alzheimer’s disease prevalence, as well as cognitive outcomes and AT(N) biomarkers across age and sex. Participants were recruited from a population-based health plan in Barcelona, Spain, and from a convenience sample recruited via services for people with intellectual disabilities in England and Scotland. They underwent assessment with the Cambridge Cognitive Examination for Older Adults with Down Syndrome, modified cued recall test and determinations of brain amyloidosis (CSF amyloid-β 42 / 40 and amyloid-PET), tau pathology (CSF and plasma phosphorylated-tau181) and neurodegeneration biomarkers (CSF and plasma neurofilament light, total-tau, fluorodeoxyglucose-PET and MRI). We used within-group locally estimated scatterplot smoothing models to compare the trajectory of biomarker changes with age in females versus males, as well as by apolipoprotein ɛ4 carriership. Our work revealed similar prevalence, age at diagnosis and Cambridge Cognitive Examination for Older Adults with Down Syndrome scores by sex, but males showed lower modified cued recall test scores from age 45 compared with females. AT(N) biomarkers were comparable in males and females. When considering apolipoprotein ɛ4, female ɛ4 carriers showed a 3-year earlier age at diagnosis compared with female non-carriers (50.5 versus 53.2 years, *P* = 0.01). This difference was not seen in males (52.2 versus 52.5 years, *P* = 0.76). Our exploratory analyses considering sex, apolipoprotein ɛ4 and biomarkers showed that female ɛ4 carriers tended to exhibit lower CSF amyloid-β 42/amyloid-β 40 ratios and lower hippocampal volume compared with females without this allele, in line with the clinical difference. This work showed that biological sex did not influence clinical and biomarker profiles of Alzheimer’s disease in adults with Down syndrome. Consideration of apolipoprotein ɛ4 haplotype, particularly in females, may be important for clinical research and clinical trials that consider this population. Accounting for, reporting and publishing sex-stratified data, even when no sex differences are found, is central to helping advance precision medicine.

## Teaser

Iulita *et al*. report that sex does not modify Alzheimer’s disease clinical outcomes and biomarker profiles in adults with Down syndrome, except for the association between sex and APOE ɛ4 on age at diagnosis. Accounting for and reporting sex-stratified data, even when no significant differences are found, is central to developing precision medicine.

## Graphical Abstract

**Graphical Abstract:**
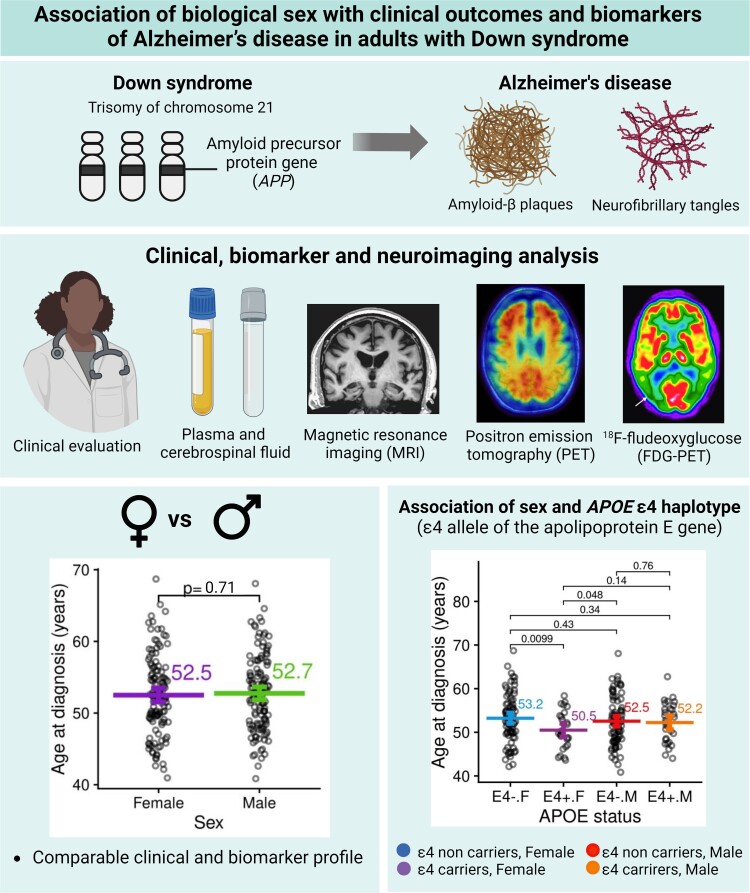

## Introduction

The topic of sex differences is now positioned as a top priority in neurology and translational neuroscience research, particularly in the context of precision medicine and personalized care strategies.^1,2^ In Alzheimer’s disease, females account for around two-thirds of patients and caregivers worldwide.^3^ While this is partly explained by differences in longevity, increasing evidence shows that biological sex can influence key aspects of the disease, including molecular pathways, cognitive progression and risk factor profiles.^4,5^

Clinically, females outperform males in verbal memory tests,^6‐8^ but this ‘female advantage’ is lost at the dementia stage,^9^ possibly due to faster rates of cognitive deterioration in females with mild cognitive impairment compared with males.^10,11^ Biomarkers including, CSF amyloid-β (Aβ)42, Aβ42/Aβ40 and amyloid-PET do not appear to differ by sex in cross-sectional studies across the Alzheimer’s disease spectrum, as reviewed by Mielke.^12^ Similarly, no sex differences in plasma and CSF phosphorylated tau at threonine 181 (p-tau181) concentrations were found,^12^ although tau-PET showed a greater extent of tau pathology in females in Alzheimer’s disease-relevant regions compared with males, even when controlling for disease severity.^13‐15^ This is in line with autopsy studies, which reported greater neurofibrillary tangle density in females.^16,17^ For neurofilament light chain (NfL), a marker of axonal damage, some studies indicate that males exhibit higher concentrations than females in CSF, but not in plasma.^18^ Evidence of sex differences in MRI biomarkers is mixed at the cross-sectional level; for a review, see Ferretti *et al*.^4^

While some biomarkers might not differ by sex, certain risk factors can impact males and females differently. For example, the ɛ4 allele of the apolipoprotein E gene (*APOE*) confers a greater risk of Alzheimer’s disease in females compared with males at ages 65–75 years.^19^ In line with this, several studies found significant interactions between sex and *APOE* ɛ4 in CSF and tau-PET biomarkers, brain hypometabolism [^18^F-fludeoxyglucose positron emission tomography (FDG-PET)] and atrophy, with females showing significantly greater pathological biomarker values at similar clinical stages.^14,20‐23^

People with Down syndrome represent the largest population with a genetic link to Alzheimer’s disease (>90% in the 7th decade).^24‐26^ Due to the trisomy of chromosome 21, which harbours the amyloid precursor protein (*APP*) gene encoding for the amyloid precursor protein, people with Down syndrome invariably develop the neuropathological changes associated with Alzheimer’s disease by middle age.^27,28^ Down syndrome, together with autosomal dominant Alzheimer's disease, constitutes the main evidence for the amyloid cascade hypothesis in which genetic mutations or gene dose loading results in increased amyloid production, leading to tau hyperphosphorylation, neurodegeneration and dementia.^29^ Indeed, the temporality of Alzheimer’s disease biomarkers in Down syndrome is similar to the sporadic forms and nearly identical to autosomal dominant Alzheimer’s disease.^25,30,31^

Despite the importance of studying Alzheimer’s disease in Down syndrome, little is known about the effect of sex on Alzheimer’s disease manifestations in this population. There are discrepant results from studies of Alzheimer’s disease risk by sex in Down syndrome,^32‐36^ and there are still no studies about sex differences across a range of biomarker modalities. Understanding the effect of sex in Down syndrome is relevant for clinical research, particularly for upcoming clinical trials on Alzheimer’s disease that consider this population.^37^

We examined the association of biological sex with clinical outcomes and biomarkers of Alzheimer’s disease in a dual-centre cohort of adults with Down syndrome. We used biochemical and neuroimaging measures to assess amyloid (A), tau (T) and neurodegeneration (N) biomarkers across age and sex. We explored whether *APOE* ɛ4 influenced the association between sex and clinical and biomarker profiles.

## Materials and methods

This study followed the STROBE reporting guidelines for cross-sectional studies.

### Study population

Participants were members of two clinical cohorts of adults with Down syndrome (*n* = 628), one from Barcelona, Spain (*n* = 584) and the other from Cambridge, UK (*n* = 44). Inclusion criteria were defined as individuals with Down syndrome of both sexes, aged 18 + years, in good general condition, who understood and accepted the procedures of the study. Exclusion criteria were inability to provide informed consent, any significant unstable medical or psychiatric disease affecting cognition, anticoagulant treatment or other blood dyscrasias that contraindicated the lumbar puncture and contraindications for MRI (claustrophobia, pacemaker, aneurysm clip and etc.) and/or pregnancy.

The study was approved by the Ethics Committee at Hospital Sant Pau (Spain), as well as by the University of Cambridge Research Ethics Committees and the Administration of Radioactive Substances Advisory Committee (UK), following the standards of the Declaration of Helsinki. In Spain, all participants or their legal representatives were required to give written informed consent. In the UK, written consent was obtained from all adults with Down syndrome who had the capacity to consent. For participants who did not have the capacity to consent, the procedures in the Mental Capacity Act of 2005 were followed. Participants were recruited between June 2009 and August 2021.

In Barcelona, study participants were part of a prospective longitudinal cohort to screen for Alzheimer’s disease in Down syndrome in Catalonia.^25^ The Cambridge cohort consisted of a convenience sample recruited via services for people with intellectual disabilities in England and Scotland.^38^ Most but not all study participants had at least one biochemical or neuroimaging biomarker assessment. A convenience sample of non-trisomic volunteers with no cognitive impairment (*n* = 173) was selected from the Sant Pau Initiative on Neurodegeneration cohort.^39^ This group was included only as a visual reference of the biomarker trajectories in euploid individuals. The reference sample had a mean age of 55.6 (9.9) years, and 61.8% (*n* = 107) were female. Differences in biomarkers between people with Down syndrome and euploid controls were described elsewhere.^25^

### Neurological and neuropsychological assessment

Intellectual disability (ID) was stratified as mild, moderate, severe or profound based on the DSM 5th Edition and using the individuals’ best level of functioning, determined from caregiver reports. Each participant further received a diagnostic evaluation of dementia in a consensus meeting between the neurologists and neuropsychologists who assessed them independently, masked to biomarker data. A diagnosis of asymptomatic was given when there was no clinical or neuropsychological suspicion of Alzheimer’s disease (i.e. absence of cognitive impairment beyond the intellectual and developmental disabilities or functional decline compared to the previous functioning). A diagnosis of prodromal Alzheimer’s disease was given when there was suspicion of Alzheimer's disease, but symptoms did not fulfill the criteria for dementia (i.e. cognitive impairment without functional changes). A diagnosis of Alzheimer’s disease dementia required evidence of cognitive impairment beyond the intellectual and developmental disabilities that interfered with everyday activities (i.e. presence of a functional decline compared to previous functioning). Functional status to differentiate prodromal and dementia stages were assessed based on anamnesis, the Dementia Questionnaire for Persons with Mental retardation and the Cambridge Examination for Mental Disorders of Older People with Down’s Syndrome and Others with Intellectual Disabilities (CAMDEX-DS). Dementia and prodromal Alzheimer’s disease were considered symptomatic Alzheimer’s disease. Our diagnostic procedures follow the recommendations of the Working Group for the Establishment of the Criteria for the Diagnosis of Dementia in Individuals with Developmental Disability.^40^

Global cognition was assessed with the Cambridge Cognitive Examination for Older Adults with Down Syndrome (CAMCOG-DS), which evaluates orientation, language, memory, attention, praxis, abstract thinking and perception.^41^ Episodic memory (immediate and delayed recall) was evaluated with the modified cued recall test (mCRT), adapted for people with intellectual disabilities.^42^ To account for the effect of ID on cognitive performance, we excluded severe or profound cases to prevent floor effects and computed *Z*-scores in the mild and moderate ID groups separately.

### Apolipoprotein E genotyping

A total of 551 participants were screened for *APOE* genotype. DNA was extracted from peripheral blood, and genotyping was determined by Sanger sequencing of the polymerase chain reaction (PCR) amplification of coding exon 4, as previously described.^43^ Individuals were dichotomized as *APOE* ɛ4 carriers or non-carriers, based on the presence of at least one ɛ4 allele.

### Plasma and CSF biomarker analyses

Blood and CSF were obtained in a subset of participants with Down syndrome and processed as previously described.^43^ CSF Aβ40 (*n* = 235), Aβ42 (*n* = 235), p-tau181 (*n* = 235) and total tau protein (t-tau, *n* = 235) were quantified with the Lumipulse automated platform (Fujirebio, Europe) following previously reported methods established in our laboratory.^44,45^ CSF NfL (*n* = 154) was measured with ELISA (UmanDx, Sweden), as previously described.^45^ Plasma p-tau181 (*n* = 514) and NfL (*n* = 499) were measured with Simoa® (Quanterix, USA), using validated assays.^25,46^

### Brain imaging

A subset of participants underwent a 3T-MRI (*n* = 243), FDG-PET (*n* = 147) and/or amyloid-PET (*n* = 119). We used the Computational Anatomy Toolbox (CAT12, http://dbm.neuro.uni-jena.de/cat) for the SPM12 software to preprocess the structural 3DT1 sequence of the MRI and extract the hippocampal and total intracranial volumes (TIVs). TIV was used to normalize differences in head size. FDG-PET images were intensity-scaled by the pons-vermis region and spatially normalized using SPM12.^39^ Standardized uptake value ratios (SUVRs) were extracted from Landau’s regions of interest.^47^ The amyloid-PET data in Barcelona was collected using ^18^F-florbetapir (2013–2017) and ^18^F-flutemetamol (2018–2021), and ^11^C-Pittsburgh compound B was used in Cambridge. Images were spatially normalized using MRI transformations computed with the Advanced Normalization Tools ^48^ and scaled using the cerebellum as the reference region.^49^ The mean cortical SUVRs were then transformed into Centiloid units.^50^

### Statistical analysis

The R software v.4.04 was used. A chi-squared test (or Fisher’s exact test when appropriate) was used to test differences between categorical data, and Mann–Whitney was used for continuous variables. Survival analysis with a log-rank test was conducted to assess sex differences in the age at the first diagnosis of symptomatic Alzheimer’s disease. Statistical significance was set at *P* < 0.05. Extreme values (>5 IQR below Q1 or above Q3) were excluded from the analysis. The final sample size for each biomarker and clinical outcome is presented in Table 1.

**Table 1:** Characteristics of the study population

		Females (*n* = 287)	Males (*n* = 341)	*P*-value
Age (years)		44.5 [33.6; 50.8]	44.0 [35.1; 50.7]	0.91
*APOE* *ɛ4* haplotype				0.63
	ɛ4 carriers	54 (21.4%)	58 (19.4%)	
	ɛ4 non-carriers	198 (78.6%)	241 (80.6%)	
Level of intellectual disability				0.09
	Mild	72 (25.7%)	61 (18.4%)	
	Moderate	144 (51.4%)	184 (55.6%)	
	Severe or profound	64 (22.9%)	86 (26.0%)	
Diagnostic group				0.78
	Asymptomatic	177 (66.0%)	218 (67.5%)	
	Symptomatic Alzheimer’s disease	91 (34.0%)	105 (32.5%)	
Medical conditions				
	Hypothyroidism (*n* = 406)	108 (58.7%)	87 (39.2%)	<0.001
	Epilepsy (*n* = 330)	17 (11.0%)	15 (8.6%)	0.58
	Sleep apnoea (*n* = 403)	15 (8.2%)	34 (15.4%)	0.04
	Depression (*n* = 433)	31 (16.0%)	36 (15.1%)	0.90
Cognition				
	CAMCOG-DS score (*n* = 441)	72.0 [56.0; 84.0]	70.5 [57.0; 83.0]	0.83
	mCRT immediate recall (*n* = 374)	35.0 [28.0; 36.0]	35.0 [31.0; 36.0]	0.69
	mCRT delayed recall (*n* = 369)	11.5 [9.0; 12.0]	11.0 [10.0; 12.0]	0.76
Fluid biomarkers				
	CSF Aβ42/Aβ40 (*n* = 235)	5.3 [4.2; 7.4] e^−2^	5.9 [4.3; 7.9] e^−2^	0.54
	CSF p-tau181 (*n* = 232)	67.0 [29.7; 153]	55.6 [29.3; 120]	0.42
	CSF total tau (*n* = 233)	519 [280; 886]	452 [256; 808]	0.53
	CSF NfL (*n* = 150)	586 [356; 876]	560 [353; 976]	0.59
	Plasma p-tau 181 (*n* = 514)	15.1 [9.4; 24.1]	12.9 [9.0; 22.2]	0.09
	Plasma NfL (*n* = 499)	12.8 [7.3; 21.8]	11.1 [6.8; 19.1]	0.20
Imaging biomarkers				
	Centiloid amyloid PET (*n* = 119)	13.9 [1.6; 59.9]	12.0 [1.7; 48.2]	0.66
	FDG-PET SUVR (*n* = 147)	1.2 [0.9; 1.4]	1.3 [1.1; 1.4]	0.40
	Hippocampal volume (*n* = 243)	5.5 [4.9; 6.0]	6.2 [5.5; 6.7]	<0.001
	Adj hippocampal volume (*n* = 243)	4.9 [4.2; 5.3] e^−3^	4.9 [4.3; 5.3] e^−3^	0.94

Unless otherwise indicated, values represent *n* (%) or median [Quartile 1 and Quartile 3]. All fluid biomarker concentration units, except for the CSF Aβ42/40 ratio, are pg/mL. *P*-values refer to analyses of chi-squared tests for categorical variables and Mann–Whitney tests for continuous variables. Aβ40, amyloid β peptide 40; Aβ42, amyloid β peptide 42; CAMCOG-DS, Cambridge Cognitive Examination for Older Adults with Down Syndrome; CSF, cerebrospinal fluid; FDG, ¹^18^F-fluorodeoxyglucose; mCRT, modified cued recall test; NfL, neurofilament light chain; PET, positron emission tomography; p-tau181, phosphorylated tau at threonine 181; SUVR, standardized uptake value ratio; TIV, total intracranial volume.

To compare changes in cognition and biomarkers across age and sex, we fitted a first-degree locally estimated scatterplot smoothing (LOESS) curve in each group independently.^51^ We used a first-order LOESS model with a tricubic weight function and a span parameter of 0.75. This approach was used to account for the non-linear trajectory of these measurements. The exact age at which the groups diverge depends on the nature of the variable, the sensitivity of the assay, the slope of the association and the sample sizes for the different measurements. We defined a clinical or biomarker difference between groups as significant at the age at which the curves diverged visually, and the 95% confidence intervals did not overlap. We also conducted independent between-group comparisons across decades using Mann–Whitney tests since most variables of interest did not follow a normal distribution, as assessed using the Shapiro–Wilk test (Supplementary material).

### Data availability

The authors may share de-identified data that underlie the results reported in this article. Data will be available upon receipt of a request detailing the study hypothesis and statistical analysis plan. All requests should be sent to the corresponding authors. The steering committee of this study will discuss all requests and decide based on the novelty and scientific rigor of the proposal whether data sharing is appropriate. All applicants will be asked to sign a data access agreement.

## Results

### Participants

A total of 628 adults with Down syndrome, including 287 (46%) females, were included (Table 1). There were no significant differences in age, proportion of *APOE* ɛ4 carriers, level of ID or prevalence of medical comorbidities by sex, except for hypothyroidism which was more common in females (58.7% versus 39.2%, *P* < 0.001). The different subsamples for each biomarker did not present differences in demographic characteristics.

When comparing recruiting sites, we found no demographic differences between the two cohorts. The core difference concerned ID (Supplementary Table 1), which is in line with the different sources of participant recruitment between the two sites (population-based versus convenience sample).

### Sex differences in Alzheimer’s disease clinical presentation

**Figure 1:**
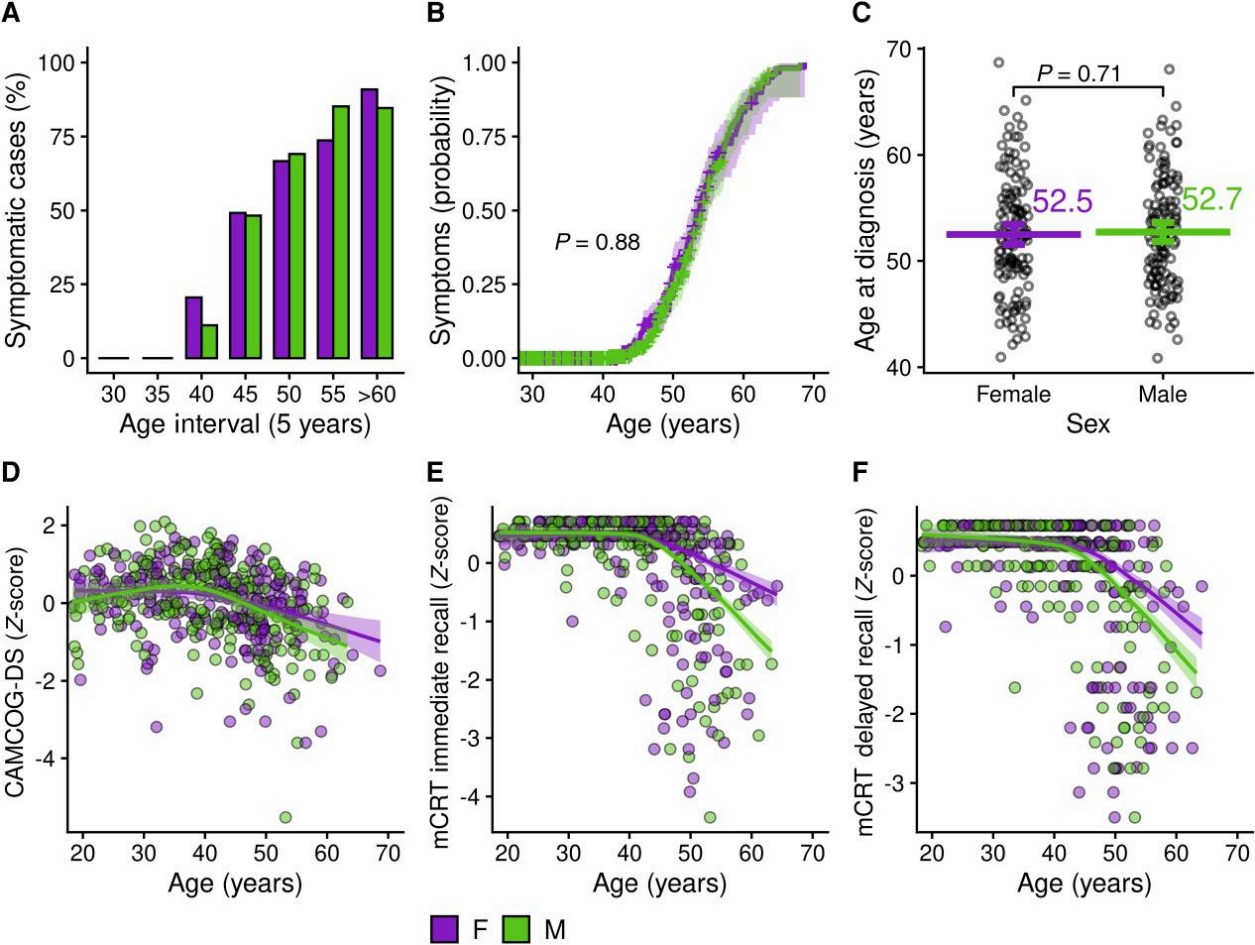
**Association of biological sex with Alzheimer’s disease prevalence and cognitive decline in adults with Down syndrome.** (**A**) Point prevalence of symptomatic Alzheimer’s disease by 5-year age intervals. Group differences were assessed with Pearson's chi-squared analysis (for details, see Supplementary Table 2). (**B**) Survival analysis showing the probability of developing symptomatic Alzheimer’s disease by age and sex. The *P*-value refers to log-rank test analysis. (**C**) Scatterplots illustrating the distribution of age at diagnosis of symptomatic Alzheimer’s disease. The graph shows the individual data points, the means and the nonparametric bootstrapped 95% confidence intervals. The *P*-value refers to a *t*-test (for details, see Supplementary Table 2). (**D**–**F**) Neuropsychological performance by age and sex at the CAMCOG-DS (**D**), immediate (**E**), and delayed (**F**) recall at the mCRT, with bands representing the 95% confidence intervals. A significant difference between LOESS curves was defined as the age at which the curves diverged visually and the 95% confidence intervals did not overlap (*P* < 0.05). CAMCOG-DS, Cambridge Cognitive Examination for Older Adults with Down Syndrome; mCRT, modified cued recall test; F, female; M, male.

We performed an age-stratified analysis to compare the prevalence of symptomatic Alzheimer’s disease between males and females, based on assessments of each participant’s first clinical visit. In both groups, the prevalence of Alzheimer’s disease increased with age, and there were no significant sex differences in prevalence across age brackets (Fig. 1A and Supplementary Table 2). A survival analysis assessing the probability of developing symptomatic Alzheimer’s disease at different ages also revealed a similar risk between males and females with Down syndrome (Fig. 1B). Finally, the age at diagnosis of Alzheimer’s disease did not differ by sex (52.5 ± 5.5 in females versus 52.7 ± 5.2 in males, *P* = 0.73, Fig. 1C and Supplementary Table 2).

We next compared CAMCOG-DS and mCRT scores between males and females by age, as age can be used as a proxy of disease progression in this population.^29^ CAMCOG-DS scores declined with age in both males and females with Down syndrome, with no apparent sex differences in the whole group (Fig. 1D and Supplementary Fig. 1) or when considering ID (Supplementary Fig. 2). For episodic memory, males presented lower mCRT scores compared with female from age 45 onwards, both for delayed and immediate recall (Fig. 1E–F and Supplementary Fig. 1).

### Sex differences in Alzheimer’s disease biomarkers

**Figure 2:**
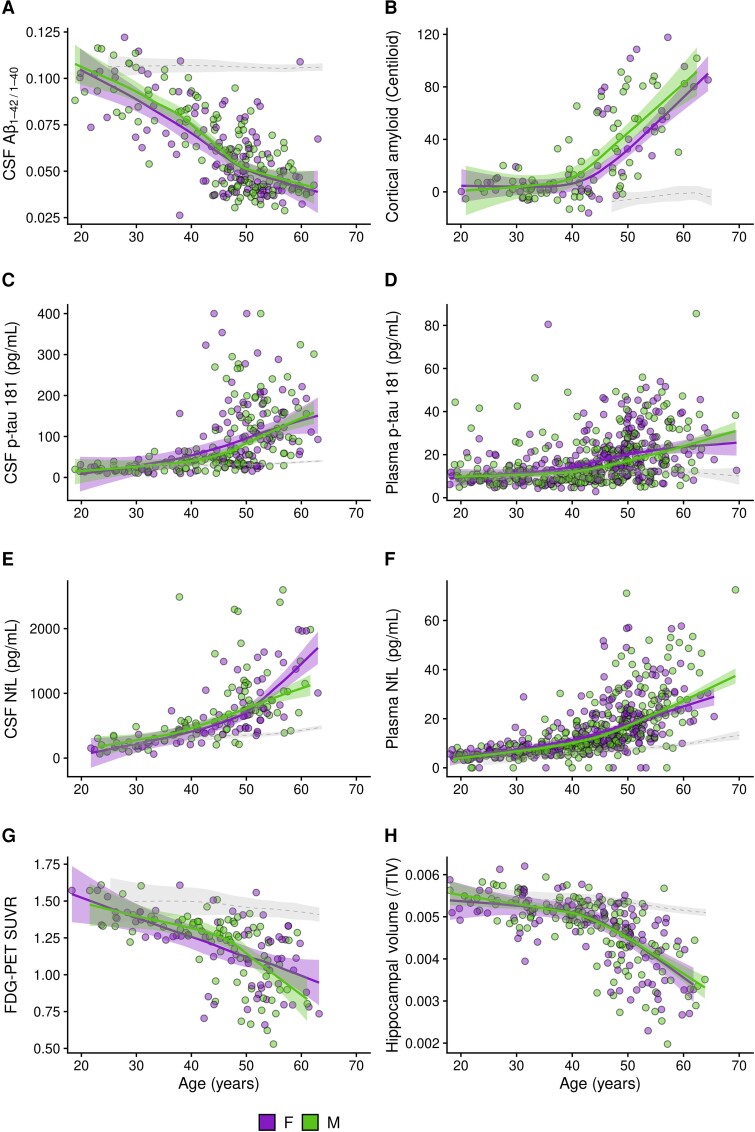
**Association of biological sex with Alzheimer’s disease biomarkers in adults with Down syndrome.** Shading represents 95% confidence intervals. The dotted lines represent the age-related changes in euploid individuals (healthy controls) for visual reference. A significant difference between LOESS curves was defined as the age at which the curves diverged visually, and the 95% confidence intervals did not overlap (*P* < 0.05). The scatterplots display, for males and females separately, the relationship between age and CSF Aβ42/40 (**A**), amyloid-PET (**B**), CSF and plasma p-tau181 (**C**, **D**), CSF and plasma NfL (**E**, **F**), FDG-PET (**G**) and hippocampal volume (**H**). Aβ40, amyloid β peptide 40; Aβ42, amyloid β peptide 42; CSF, cerebrospinal fluid; FDG, ¹^18^F-fluorodeoxyglucose; NfL, neurofilament light chain; SUVR, standardized uptake value ratio; TIV, total intracranial volume; F, female; M, male.

The CSF Aβ42/Aβ40 ratio (Fig. 2A, Supplementary Fig. 3 and Supplementary Table 3) and Aβ42 (Supplementary Fig. 4 and Supplementary Table 3) declined with age with no significant differences between males and females with Down syndrome. CSF concentrations of Aβ40 did not change significantly with age and were comparable between males and females (Supplementary Fig. 4 and Supplementary Table 3). We also found comparable increases in cortical amyloid burden (as assessed by amyloid-PET) with age by sex (Fig. 2B, Supplementary Fig. 3 and Supplementary Table 3).

There were no significant differences in plasma or CSF p-tau181 concentrations between males and females. However, between the ages of 40 and 50 years, female showed a trend towards higher p-tau181 concentrations in CSF and plasma (Fig. 2C–D and Supplementary Fig. 3 and Supplementary Table 3 for the analysis by decades). No other trends or differences were seen at other age intervals.

We next compared biomarkers of neurodegeneration. Males and females showed overall similar increases in CSF and plasma NfL with age (Fig. 2E–F, Supplementary Fig. 3 and Supplementary Table 3). The CSF and plasma NfL LOESS curves only tended to diverge between sexes at the older ages (55–65 years). There were no sex differences in the trajectories of CSF t-tau in CSF (Supplementary Fig. 4 and Supplementary Table 3). For FDG-PET, the glucose metabolism in typical Alzheimer’s disease brain regions decreased similarly with age in both males and females with Down syndrome (Fig. 2G, Supplementary Fig. 3 and Supplementary Table 3). Likewise, hippocampal volumes, when adjusted by total intracranial volume, decreased similarly with age in males and females (Fig. 2H, Supplementary Fig. 3 and Supplementary Table 3).

### Association of sex and APOE ɛ4 haplotype in Down syndrome

**Figure 3:**
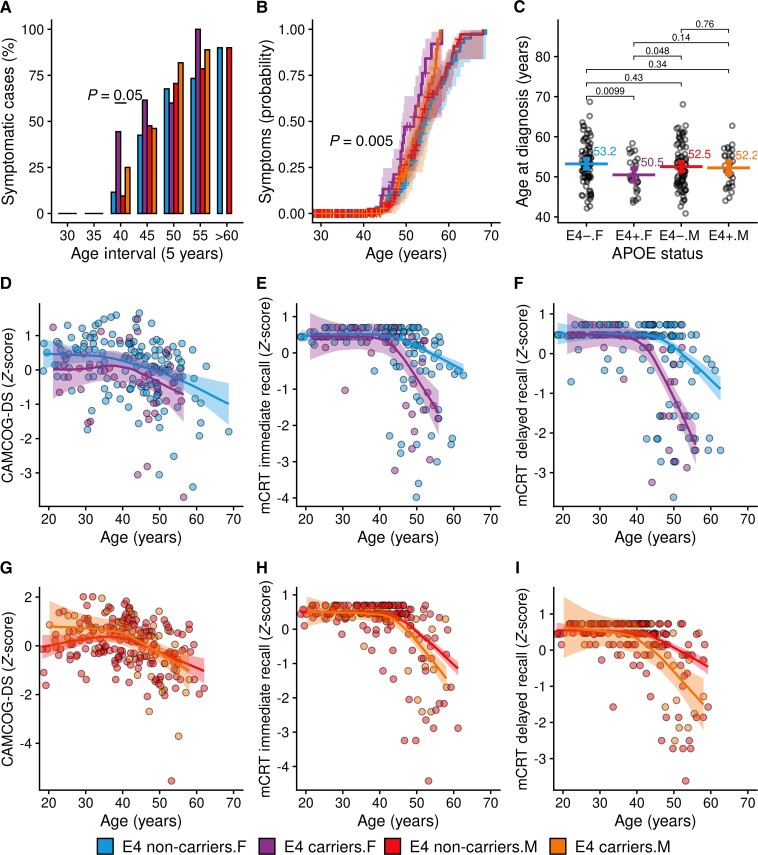
**Association of *APOE* ɛ4 haplotype and biological sex with Alzheimer’s disease prevalence and cognitive decline.** (**A**) Point prevalence of symptomatic Alzheimer’s disease by sex and *APOE* ɛ4 haplotype across 5-year age intervals. Group differences were assessed with Pearson's chi-squared analysis (for details, see Supplementary Table 4). (**B**) Survival analysis showing the probability to develop symptomatic Alzheimer’s disease by sex and *APOE* haplotype across age. The *P*-value refers to log-rank test analysis. (**C**) Scatterplots illustrating the distribution of age at diagnosis of Alzheimer’s disease and showing the individual data points, the means and the nonparametric bootstrapped 95% confidence intervals. The *P*-values refer to *t*-tests (for details, see Supplementary Table 4). (**D**–**I**) Age-related changes in neuropsychological performance at the CAMCOG-DS (**D**, **G**), immediate (**E**, **H**) and delayed recall (**F**, **I**) at the mCRT, with bands representing the 95% confidence intervals in male and female separately. A significant difference between LOESS curves was defined as the age at which the curves diverged visually and the 95% confidence intervals did not overlap (*P* < 0.05). CAMCOG-DS, Cambridge Cognitive Examination for Older Adults with Down Syndrome; mCRT, modified cued recall test; F, female; M, male; ɛ4, *APOE* ɛ4 carriers.

When we considered the *APOE* ɛ4 haplotype, we found that the prevalence of symptomatic Alzheimer’s disease was different across groups at age 40–45 years (Fig. 3A, Supplementary Table 4, *P* = 0.05), with the greatest prevalence in female ɛ4 carriers (44.4% versus 11.5% for female non-carriers, 9.5% for male non-carriers and 25% for male ɛ4 carriers). Likewise, the probability of developing symptoms of Alzheimer’s disease at younger ages was highest for females with an *APOE* ɛ4 allele (*P* = 0.005, Fig. 3B). In addition, female ɛ4 carriers were diagnosed with symptomatic Alzheimer’s disease on average 3 years earlier than female without this allele (50.5 versus 53.2 years, *P* = 0.01, Fig. 3C and Supplementary Table 4). For males, there was no difference in age at diagnosis between ɛ4 carriers and non-carriers (52.2 versus 52.5 years, *P* = 0.76).

When we explored the association between sex and *APOE* ɛ4 on cognitive performance, we found that *APOE* ɛ4 was associated with poorer episodic memory in both sexes, but not with global cognition (CAMCOG-DS scores). The difference in mCRT scores between ɛ4 carriers and non-carriers was more pronounced in females compared with males (Fig. 3 E–F and H–I).

**Figure 4:**
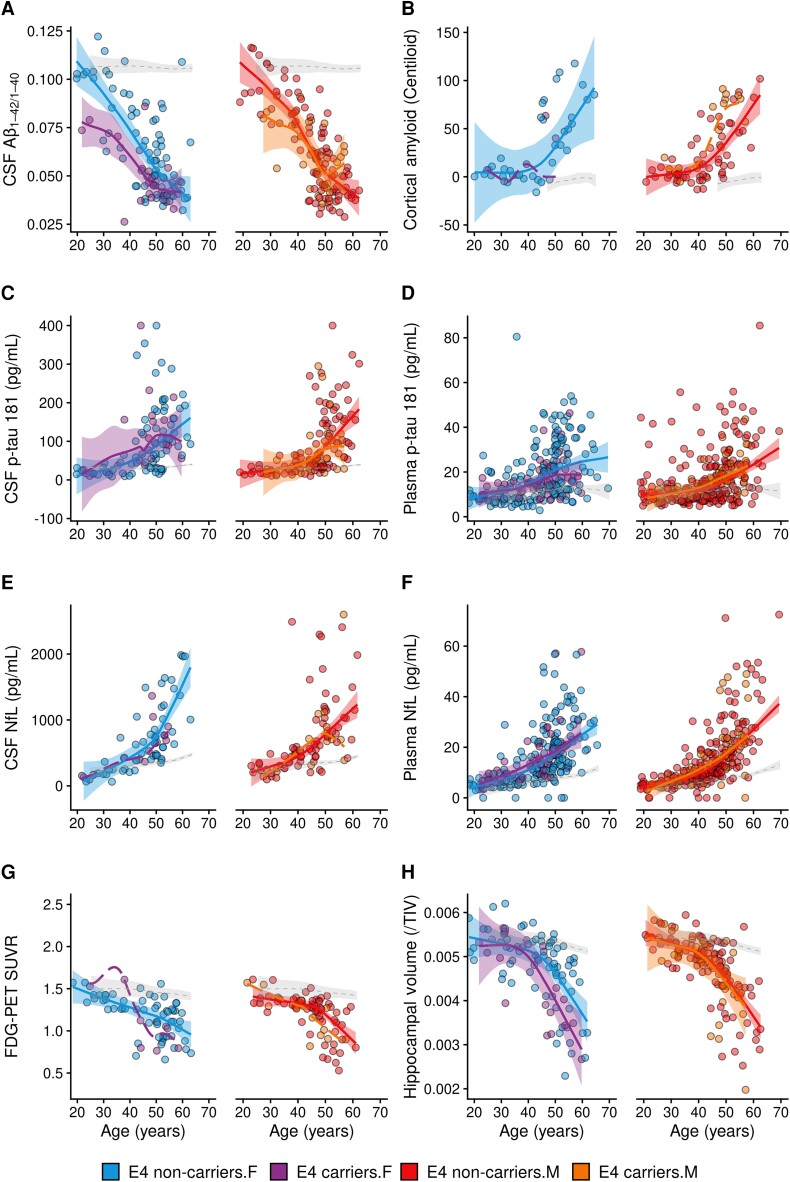
**Association of *APOE* ɛ4 haplotype and sex with Alzheimer’s disease biomarkers in adults with Down syndrome.** Shading represents 95% confidence intervals. The dotted lines represent the age-related changes in euploid individuals for visual reference. The dashed line without confidence intervals was used in groups with a sample size inferior to 20 participants. A significant difference between LOESS curves was defined as the age at which the curves diverged visually and the 95% confidence intervals did not overlap (*P* < 0.05). The scatterplots display, for males and females separately, the relationship between age and APOE ε4 carriership with CSF Aβ42/40 (**A**), amyloid-PET (**B**), CSF and plasma p-tau181 (**C, D**), CSF and plasma NfL (**E, F**), FDG-PET (**G**) and hippocampal volume (**H**). Aβ40, amyloid β peptide 40; Aβ42, amyloid β peptide 42; *APOE*, apolipoprotein E; CSF, cerebrospinal fluid; FDG, ¹^18^F-fluorodeoxyglucose; NfL, neurofilament light chain; SUVR, standardized uptake value ratio; TIV, total intracranial volume; F, female; M, male; ɛ4, *APOE* ɛ4 carriers.

At the level of biomarkers, we performed an exploratory analysis stratifying by sex and *APOE* ɛ4 haplotype, with the limitation that the sample sizes of some biomarker subgroups were small (<20). We found that female ɛ4 carriers had lower levels of the CSF Aβ42/Aβ40 ratio between ages 20 and 50 years, overlapping afterward (Fig. 4A). In males, ɛ4 carriers tended to show earlier increases in amyloid-PET uptake compared with non-carriers (Fig. 4B). The sample size of the female ɛ4 subgroup for amyloid-PET was too small for comparisons. Both males and females tended to show lower cerebral glucose metabolism between ages 40 and 50 years in ɛ4 carriers compared with non-carriers (Fig. 4G). Hippocampal volumes also tended to be lower in female ɛ4 carriers between ages 45 and 60 years, compared with females who did not carry this allele (Fig. 4H). No other relevant sex differences in Alzheimer’s disease biomarkers were detected after stratification by *APOE* ɛ4 haplotype in this population (Fig. 4). Supplementary Table 5 and Supplementary Table 6 detail the relevant demographic, clinical and biomarker outcomes and sample size of the subgroups included in the exploratory analyses segregated by *APOE* ɛ4 carriership and sex.

## Discussion

Growing evidence is pointing to sex differences in Alzheimer’s disease, including clinical features, disease course and pathology burden.^4,52^ Whether these differences are consistent across Alzheimer’s disease populations, including the genetic forms, is not yet well understood.

We addressed this by examining a large clinical cohort of adults with Down syndrome. We found that males and females with Down syndrome showed similar clinical presentation and comparable Alzheimer’s disease biomarker profiles. Despite these similarities, we did observe better episodic memory in females from age 45 onwards, and a clear *APOE* ɛ4 and sex association on disease onset.

Previous studies assessing sex differences in age at diagnosis or Alzheimer’s disease risk in Down syndrome reported conflicting findings. Some have found greater risk and earlier age at diagnosis in males,^32,36^ while others have found greater risk or earlier age at diagnosis in females,^33,53^ trends in such direction but no significance^35^ or no sex differences after adjusting for confounding variables.^34^ The reasons for these inconsistencies might be related to differences in study design, with some studies using longitudinal, prospective, clinician-driven diagnosis of dementia at recurrent follow-ups, while others reviewing medical records for previous dementia diagnoses which could be potentially biased by differences in referral to a diagnostic physician. Moreover, the age range of studies included individuals between 30 and 70 years, but overall sample sizes varied from *n* = 21 to *n* = 100 or even over *n* = 400. The distribution of intellectual disability was also heterogeneous. Likewise, different proportions of *APOE* ɛ4 carriers or of females in menopause, perimenopause and post-menopause, which are known factors that can impact age at diagnosis,^54^ might help explain these conflicting findings.

We found overlapping trajectories of amyloid and neurodegeneration biomarkers between males and females with Down syndrome explored across different modalities (CSF, plasma and PET), indicating no main effect of sex on these biomarkers in this population. This is in line with the general literature on sporadic Alzheimer’s disease, where no clear effect of sex on global amyloid burden has been reported.^15,55,56^ Contrary to some studies in the general population,^18^ males with Down syndrome did not show higher NfL concentrations in CSF. The higher levels of NfL in healthy males might be attributed to the greater proportion of white matter compared with females, as reported by some studies.^57^ It would be interesting to investigate whether sex differences in white matter integrity or regional variations exist in Down syndrome that would help interpret this result.

The concentrations of plasma and CSF p-tau181 were also highly overlapping across both sexes. Although this suggests a similar degree of tau pathology, we cannot rule out the existence of regional differences, as our study did not include tau-PET imaging data. It would be interesting to examine this, since an autopsy study found greater neurofibrillary tangle density in females with Down syndrome compared with males,^53^ and there is limited information from other neuropathology studies due to the absence of reporting of sex-stratified data^28^ or insufficient cases to allow for comparisons.^58‐61^

While we found similar ages at diagnosis of Alzheimer’s disease between sexes and comparable scores on global cognition, we noted that males exhibited lower mCRT scores from age 45 compared with females. Because of the ceiling effect on mCRT scores observed in our study before this age, we cannot rule out that males and females experience a similar decline in mCRT scores in Down syndrome, with females starting at a higher level at younger ages. Another possibility is that females manifest cognitive resilience to Alzheimer’s disease neuropathological changes. Vila-Castelar *et al*.^62^ have similarly reported poorer verbal episodic memory performance in male carriers of the *PSEN1* E280A variant at the asymptomatic stage, with no sex differences in hippocampal volume. These and our results show concordance between two genetically-at risk Alzheimer’s disease populations. Studies that support the resilience hypothesis have also found higher cortical thickness or better verbal memory scores in females compared with males at similar levels of tau pathology within the sporadic Alzheimer’s disease spectrum.^8,63^

Our study also showed that even if the main effect of sex on Alzheimer’s disease biomarkers was subtle or null, sex can interact with other factors, and thus impact the clinical expression of Alzheimer’s disease in Down syndrome. We found that both sexes were vulnerable to the genetic risk of *APOE* ɛ4, as we recently reported,^64^ but that females with Down syndrome showed earlier age at diagnosis by 3 years. This difference was not seen in males. Our exploratory analyses considering sex, *APOE* ɛ4 and biomarker profiles showed that female ɛ4 carriers exhibited lower CSF Aβ42/Aβ40 ratio and lower hippocampal volume compared with females without this allele, in line with the clinical difference. Given that sample sizes after stratification were small, these exploratory results need further confirmation in other cohorts.

Male ɛ4 carriers tended to show earlier increases in amyloid-PET uptake compared with male non-carriers, but we cannot rule out a similar response in females given the small sample size of this subgroup. Indeed, a study by Cacciaglia *et al*.^65^ recently showed that age, *APOE* ɛ4 and female sex were associated with higher amyloid-PET uptake in posterior middle cortical regions in cognitively unimpaired individuals. Likewise, *APOE* ɛ4 carriers in both sexes tended to show lower cerebral glucose metabolism compared with non-carriers, although the sample size of the subgroups was small to compare by sex. Studies in the general population indicate that female *APOE* ɛ4 carriers manifest widespread clusters of hypometabolism, while males show an isolated cluster of hypometabolism in the precuneus compared with non-carriers.^20^ Moreover, a recent study analysing longitudinal data from ADNI showed that *APOE* ɛ4 status and sex modified the progression of disease (from pre-clinical to dementia stages), with female *APOE* ɛ4 carriers showing the fastest cognitive decline.^66^ The *APOE ɛ4-*sex association is also supported by single-nucleus transcriptome studies where differential transcriptional responses to Alzheimer’s disease neuropathology were shown in males and females.^67^ This suggests that consideration of *APOE ɛ4* haplotype and sex may have relevant pathophysiological implications in Alzheimer’s disease, both in sporadic and genetically determined populations.

Several mechanisms driving sex differences in Alzheimer’s disease have been considered, including the drop of sex hormones with menopause, sex differences in the manifestation of cerebrovascular and cardiovascular pathologies as well as in immune system responses.^4^ A recent review by Andrews and colleagues, discusses such mechanisms in the context of Down syndrome.^68^ Females with Down syndrome experience menopause ∼5–7 years earlier than the general population.^69,70^ Oestrogens (the primary female sex hormones) act through receptors that are also present in the brain^71^ and have been reported to reduce Aβ toxicity by stimulating the metabolism of APP, inhibiting the aggregation of Aβ, or stimulating its degradation.^72‐74^ Besides modulating amyloid pathology, oestradiol (the primary form of oestrogen) is known to promote cholinergic activity^75^ as well as to enhance memory consolidation and synaptic plasticity in transgenic mice.^76^ Interestingly, this protective effect is not seen in mice who are *APOE* ɛ4 carriers.^76^ Given the neuroprotective functions of oestrogens and their drop in menopause, it has been postulated that such event may contribute to the damaging effects on cognition and higher vulnerability to Alzheimer’s disease in females.^77,78^ Indeed, studies in clinical cohorts of females with Down syndrome have reported significant associations between age at menopause,^54,70,79^ differences in bioavailable oestrogen levels^80^ and polymorphisms in oestrogen receptors^81^ and age at diagnosis of dementia.

Outside the context of Alzheimer’s disease, sex differences in Down syndrome have also been studied. De Gonzalo-Calvo *et al*.^82^ found sex-related differences in biochemical and haematological parameters in a clinical cohort of adults with Down syndrome. Further, Startin *et al*.^83^ reported sex differences in the prevalence of comorbidities known to occur commonly in people with Down syndrome, finding higher prevalence rates of otitis and reflux in males and higher hypothyroidism in females, in line with our results. Both studies outlined recommendations for the incorporation of such knowledge into clinical practice, highlighting that the study of sex differences is relevant for and beyond Alzheimer’s disease.

Our study has strengths and limitations. We recruited the largest clinical cohort of adults with Down syndrome with clinical and multimodal biomarker assessments (plasma, CSF, MRI and PET) to compare clinical and biomarker trajectories between males and females. Its limitations include the lack of tau-PET imaging, its cross-sectional design and the relatively small sample size for some biomarkers, especially for the investigation of sex differences across *APOE* ɛ4 haplotype (e.g. FDG-PET and amyloid-PET in females). Nevertheless, the uniform development of Alzheimer’s disease pathology in Down syndrome and its consistent age at onset^29,84^ allow quasi-longitudinal studies in this population. Of note, our study did not consider menopause or hormonal status, which can impact risk in a sex-specific manner.^54,80,85,86^ We also could not conduct sub-analysis by race, as DABNI is a population-based cohort reflecting the population of Catalonia, which is predominantly White.

Taken together, our results indicate that sex does not modify Alzheimer’s disease clinical outcomes and biomarker trajectories in adults with Down syndrome, except for the association between sex and *APOE ɛ4* haplotype. In light of the growth of personalized healthcare, we urge the scientific and medical community to account for, report and publish sex-stratified data, even when no sex differences are found.

## Supplementary Material

fcad074_Supplementary_Data

## Abbreviations

APOE =: apolipoprotein E
APP =: amyloid precursor protein
Aβ =: amyloid-β
CAMCOG-DS =: Cambridge Cognitive Examination for Older Adults with Down Syndrome
CAMDEX-DS =: Cambridge Examination for Mental Disorders of Older People with Down’s Syndrome and Others with Intellectual Disabilities
FDG-PET =: ^18^F-fludeoxyglucose positron emission tomography
ID =: intellectual disability
LOESS =: locally estimated scatterplot smoothing
mCRT =: modified cued recall test
NfL =: neurofilament light chain
p-tau181 =: phosphorylated tau at threonine 181
PSEN1 =: presenilin 1
SUVR =: standardized uptake value ratio
t-tau =: total tau
TIV =: total intracranial volume
