# Supplementary material for: Association of biological sex with clinical outcomes and biomarkers of Alzheimer’s disease in adults with Down syndrome
Source: Brain Commun. 2023 Mar 17;5(2):fcad074. doi: 10.1093/braincomms/fcad074 (PMC10088472; doi:10.1093/braincomms/fcad074)
Supplement: fcad074_Supplementary_Data [file fcad074_supplementary_data.docx]

**Supplementary Information**

**eMethods and Results**

**Table of Contents**

[Supplementary Table 1. Demographic data for each recruiting site. 2](#_Toc121302676)

[Supplementary Table 2. Detailed data and statistical values for Figure 1. 3](#_Toc121302677)

[Supplementary Table 3. Detailed data and statistical values for Supplementary Fig. 1, 3-4. 4](#_Toc121302678)

[Supplementary Table 4. Detailed data and statistical values for Figure 3. 8](#_Toc121302679)

[Supplementary Table 5: Characteristics of the study population used in the exploratory analyses assessing the interaction between sex and APOE ε4. 9](#_Toc121302680)

[Supplementary Table 6: Sample size for the exploratory analyses assessing the interaction between sex and APOE ε4 status. 11](#_Toc121302681)

[Supplementary Fig. 1. Association between biological sex and cognitive performance analyzed by decades in adults with Down syndrome. 12](#_Toc121302682)

[Supplementary Fig. 2. Association between biological sex and cognitive performance in adults with Down syndrome with mild and moderate intellectual disability. 13](#_Toc121302683)

[Supplementary Fig. 3. Association between biological sex and Alzheimer’s disease biomarkers in adults with Down syndrome. 14](#_Toc121302684)

[Supplementary Fig. 4. Association between biological sex and CSF levels of Aß_1-42_, Aß_1-40_, and total tau in adults with Down syndrome. 15](#_Toc121302685)

# Supplementary Table 1. Demographic data for each recruiting site.

|  |  | **Barcelona**  **(*n* = 584)** | **Cambridge**  **(*n* = 44)** | **p value** | ***n*** |
| --- | --- | --- | --- | --- | --- |
|  |  |  |  |  |  |
| **Age (years)** | | 44.5 [34.7; 50.9] | 42.1 [34.1; 48.8] | 0.50 | 628 |
| **Sex, *n* (%)** | |  |  | 0.61 | 628 |
|  | Female | 269 (46.1%) | 18 (40.9%) |  |  |
|  | Male | 315 (53.9%) | 26 (59.1%) |  |  |
| ***APOE* ɛ4 status, *n* (%)** | |  |  | 0.35 | 551 |
|  | ɛ4 carriers | 102 (19.8%) | 10 (27.8%) |  |  |
|  | Non-Carriers | 413 (80.2%) | 26 (72.2%) |  |  |
| **Alzheimer’s disease symptoms, *n* (%)** | |  |  | 0.55 | 591 |
|  | Asymptomatic | 364 (66.4%) | 31 (72.1%) |  |  |
|  | Symptomatic AD | 184 (33.6%) | 12 (27.9%) |  |  |
| **Medical conditions, *n* (%)** | |  |  |  |  |
|  | Hypothyroidism | 176 (47.8%) | 19 (50.0%) | 0.93 | 406 |
|  | Epilepsy | 32 (10%) | 0 (0.0%) | 0.61 | 330 |
|  | Ophthalmic problem | 232 (66.7%) | 28 (71.8%) | 0.64 | 387 |
|  | Auditive problem | 51 (12.7%) | 13 (33.3%) | 0.001 | 441 |
|  | Depression | 67 (17.0%) | 0 (0.0%) | 0.010 | 433 |
| **Level of intellectual disability, *n* (%)** | |  |  | <0.001 | 489 |
|  | Mild | 117 (20.4%) | 16 (42.1%) |  |  |
|  | Moderate | 306 (53.4%) | 22 (57.9%) |  |  |
|  | Severe or profound | 150 (26.2%) | 0 (0.0%) |  |  |
| **Cognition** | |  |  |  |  |
|  | CAMCOG-DS score | 65.0 [49.0; 81.0] | 77.0 [63.2; 89.5] | 0.001 | 489 |
|  | CAMCOG-DS (mild/moderate ID) | 71.0 [56.0; 83.0] | 77.0 [63.2; 89.5] | 0.023 | 417 |
|  |  |  |  |  |  |

*Unless otherwise indicated, values are n (%) or median [Quartile 1; Quartile 3]. Level percentages for intellectual disability (ID) and diagnostic group were calculated according to the total of patients with available data in each group. P-values refer to analyses of chi-squared tests for categorical variables and Mann-Whitney tests for continuous variables. Abbreviations: AD = Alzheimer’s disease; CAMCOG-DS = Cambridge Cognitive Examination for Older Adults with Down syndrome; ID = intellectual disability.*

# Supplementary Table 2. Detailed data and statistical values for Figure 1.

| **Figure 1a** | |  |  |  |
| --- | --- | --- | --- | --- |
| **Age range** | **Sex** | **N** | **Frequency Symptomatic cases** | **Statistic (chi-squared test)** |
| 30-35 | F | 28 | 0 | na |
|  | M | 31 | 0 |  |
| 35-40 | F | 24 | 0 | na |
|  | M | 40 | 0 |  |
| 40-45 | F | 39 | 20.5 | X2 (1, N = 93) = 0.92,p=0.34 |
|  | M | 54 | 11.1 |  |
| 45-50 | F | 61 | 49.2 | X2 (1, N = 119) = 0,p=1.00 |
|  | M | 58 | 48.3 |  |
| 50-55 | F | 48 | 66.7 | X2 (1, N = 103) = 0,p=0.96 |
|  | M | 55 | 69.1 |  |
| 55-60 | F | 19 | 73.7 | X2 (1, N = 46) = 0.35,p=0.55 |
|  | M | 27 | 85.2 |  |
| >60 | F | 11 | 90.9 | X2 (1, N = 24) = 0,p=1.00 |
|  | M | 13 | 84.6 |  |
|  |  |  |  |  |
| **Figure 1c** | |  |  |  |
|  | **Sex** | **N** | **Mean (SD)** | **Statistic (two-sample T-test)** |
|  | F | 125 | 52.5 (5.5) | t(248.11) = -0.37, p =0.72, d = -0.05 |
|  | M | 126 | 52.7 (5.2) |  |

*Abbreviations: F = Female; M = Male.*

# Supplementary Table 3. Detailed data and statistical values for Supplementary Fig. 1, 3-4.

| **Supplementary Figure 1a (CAMCOG-DS Total)** | | | | |
| --- | --- | --- | --- | --- |
| **Decade** | **Sex** | **N** | **Median (IQR)** | **Statistic (Mann-Whitney)** |
| 20s | F | 35 | 0.5 (0.6) | U=794, p=0.23 |
|  | M | 39 | 0.1 (1) |  |
| 30s | F | 45 | 0.2 (1.3) | U=1253, p=0.31 |
|  | M | 63 | 0.5 (1.1) |  |
| 40s | F | 80 | -0.1 **(**1.2**)** | U=2701.5, p=0.04 |
|  | M | 83 | 0.3 (1.3) |  |
| 50s | F | 41 | 0 (0.8) | U=1204.5, p=0.03 |
|  | M | 46 | -0.6 **(**1.3**)** |  |
| >60 | F | 6 | -1.4 **(**0.7**)** | U=11, p=0.71 |
|  | M | 3 | -1.7 **(**1**)** |  |
|  | | | | |
| **Supplementary Figure 1b (mCRT Immediate Recall)** | | | | |
| **Decade** | **Sex** | **N** | **Median (IQR)** | **Statistic (Mann-Whitney)** |
| 20s | F | 34 | 0.5 (0.1) | U=542.5, p=0.23 |
|  | M | 38 | 0.5 (0.2) |  |
| 30s | F | 34 | 0.5 (0.2) | U=845.5, p=0.97 |
|  | M | 50 | 0.5 (0.4) |  |
| 40s | F | 67 | 0.5 (1) | U=1971.5, p=0.14 |
|  | M | 69 | 0.5 (0.5) |  |
| 50s | F | 41 | -0.4 **(**1.9**)** | U=822.5, p=0.28 |
|  | M | 35 | -0.9 **(**1.9**)** |  |
| >60 | F | 4 | -0.5 **(**0.1**)** | U=8, p=0.13 |
|  | M | 2 | -2.3 **(**0.6**)** |  |
|  | | | | |
| **Supplementary Figure 1c (mCRT Delayed Recall)** | | | | |
| **Decade** | **Sex** | **N** | **Median (IQR)** | **Statistic (Mann-Whitney)** |
| 20s | F | 33 | 0.5 (0) | U=514, p=0.22 |
|  | M | 37 | 0.5 (0.2) |  |
| 30s | F | 36 | 0.5 (0.3) | U=903, p=0.77 |
|  | M | 52 | 0.5 (0.6) |  |
| 40s | F | 67 | 0.4 (0.7) | U=1973.5, p=0.28 |
|  | M | 66 | 0.4 (0.6) |  |
| 50s | F | 38 | -0.7 **(**2.5**)** | U=738, p=0.42 |
|  | M | 35 | -1 **(**1.8**)** |  |
| >60 | F | 4 | -0.3 (0.7) | U=3, p=0.80 |
|  | M | 1 | -1.7 **(**0**)** |  |
|  | | | | |
| **Supplementary Figure 3a (CSF Aß42/40)** | | | | |
| **Decade** | **Sex** | **N** | **Median (IQR)** | **Statistic (Mann-Whitney)** |
| 20s | F | 10 | 0.101 (0.019) | U=56, p=0.44 |
|  | M | 14 | 0.1 (0.022) |  |
| 30s | F | 14 | 0.089 (0.035) | U=141, p=0.79 |
|  | M | 19 | 0.082 (0.016) |  |
| 40s | F | 40 | 0.053 (0.022) | U=972, p=0.70 |
|  | M | 51 | 0.059 (0.028) |  |
| 50s | F | 37 | 0.043 (0.014) | U=825, p=0.92 |
|  | M | 44 | 0.044 (0.016) |  |
| >60 | F | 4 | 0.039 (9e-03) | U=4, p=1.00 |
|  | M | 2 | 0.04 (3e-03) |  |
|  | | | | |
| **Supplementary Figure 3b (Centiloid)** | | | | |
| **Decade** | **Sex** | **N** | **Median (IQR)** | **Statistic (Mann-Whitney)** |
| 20s | F | 7 | 7.2 (6.9) | U=34, p=0.54 |
|  | M | 8 | 4.4 (8.8) |  |
| 30s | F | 10 | 1.7 (6.6) | U=94, p=0.66 |
|  | M | 21 | 2.3 (12.6) |  |
| 40s | F | 14 | 17.3 (63.1) | U=208, p=0.84 |
|  | M | 31 | 14.7 (48.1) |  |
| 50s | F | 10 | 52.4 (49.9) | U=69, p=0.83 |
|  | M | 13 | 56 (35.3) |  |
| >60 | F | 3 | 85.3 (7.7) | U=2, p=0.80 |
|  | M | 2 | 92.1 (9.7) |  |
|  | | | | |
| **Supplementary Figure 3c (CSF p-tau 181)** | | | | |
| **Decade** | **Sex** | **N** | **Median (IQR)** | **Statistic (Mann-Whitney)** |
| 20s | F | 10 | 16.9 (11.2) | U=58.5, p=0.52 |
|  | M | 14 | 20.5 (11.1) |  |
| 30s | F | 14 | 20.6 (27.8) | U=108.5, p=0.38 |
|  | M | 19 | 27.9 (30) |  |
| 40s | F | 39 | 67 (97.7) | U=1188.5, p=0.08 |
|  | M | 50 | 48.3 (78) |  |
| 50s | F | 36 | 106.4 (113.8) | U=765, p=0.80 |
|  | M | 44 | 116.7 (95.4) |  |
| >60 | F | 4 | 151.8 (93.7) | U=2, p=0.53 |
|  | M | 2 | 228.3 (72.8) |  |
|  | | | | |
| **Supplementary Figure 3d (plasma p-tau 181)** | | | | |
| **Decade** | **Sex** | **N** | **Median (IQR)** | **Statistic (Mann-Whitney)** |
| 20s | F | 37 | 9.1 (3.6) | U=677, p=0.65 |
|  | M | 39 | 10.2 (5.6) |  |
| 30s | F | 41 | 8.7 (8.4) | U=1165, p=0.66 |
|  | M | 60 | 10.5 (5.5) |  |
| 40s | F | 81 | 16.1 (10.9) | U=5123, p=0.01 |
|  | M | 104 | 14 (9.7) |  |
| 50s | F | 65 | 22.7 (21.4) | U=2412, p=0.29 |
|  | M | 67 | 21.7 (16.2) |  |
| >60 | F | 10 | 21.1 (11.4) | U=39, p=0.44 |
|  | M | 10 | 28.1 (19.7) |  |
|  | | | | |
| **Supplementary Figure 3e (CSF NfL)** | | | | |
| **Decade** | **Sex** | **N** | **Median (IQR)** | **Statistic (Mann-Whitney)** |
| 20s | F | 8 | 160.1 (50.2) | U=11, p=0.01 |
|  | M | 9 | 247.8 (125.8) |  |
| 30s | F | 11 | 247.1 (173.8) | U=68, p=0.17 |
|  | M | 18 | 360 (219.5) |  |
| 40s | F | 23 | 626.7 (334.5) | U=344, p=0.84 |
|  | M | 31 | 559.5 (623.3) |  |
| 50s | F | 22 | 794.7 (739.3) | U=235, p=0.69 |
|  | M | 23 | 823 (384.3) |  |
| >60 | F | 3 | 1963 (479.7) | U=2, p=0.80 |
|  | M | 2 | 1568 (416.5) |  |
| **Supplementary Figure 3f (Plasma NfL)** | | | | |
| **Decade** | **Sex** | **N** | **Median (IQR)** | **Statistic (Mann-Whitney)** |
| 20s | F | 38 | 5.8 (2.7) | U=820, p=0.22 |
|  | M | 37 | 5.3 (1.9) |  |
| 30s | F | 39 | 7.2 (4.1) | U=1171.5, p=0.90 |
|  | M | 61 | 7.5 (3.7) |  |
| 40s | F | 82 | 14.6 (11.3) | U=4855.5, p=0.04 |
|  | M | 101 | 12.6 (7.9) |  |
| 50s | F | 61 | 21.6 (16.3) | U=1916.5, p=0.75 |
|  | M | 65 | 22.4 (13.6) |  |
| >60 | F | 7 | 24.1 (10) | U=7, p=0.01 |
|  | M | 8 | 44 (13.9) |  |
|  | | | | |
| **Supplementary Figure 3g (FDG-PET)** | | | | |
| **Decade** | **Sex** | **N** | **Median (IQR)** | **Statistic (Mann-Whitney)** |
| 20s | F | 8 | 1.4 (0.2) | U=46, p=0.37 |
|  | M | 9 | 1.4 (0.2) |  |
| 30s | F | 12 | 1.3 (0.2) | U=54, p=0.49 |
|  | M | 11 | 1.4 (0.1) |  |
| 40s | F | 12 | 1.3 (0.3) | U=198, p=0.68 |
|  | M | 36 | 1.3 (0.2) |  |
| 50s | F | 33 | 1.1 (0.3) | U=407, p=0.46 |
|  | M | 22 | 1 (0.4) |  |
| >60 | F | 3 | 0.8 (0.1) | U=1, p=1.00 |
|  | M | 1 | 0.8 (0) |  |
|  | | | | |
| **Supplementary Figure 3h (Adjusted hippocampal volume)** | | | | |
| **Decade** | **Sex** | **N** | **Median (IQR)** | **Statistic (Mann-Whitney)** |
| 20s | F | 13 | 0.0053 (5.2e-04) | U=89, p=0.53 |
|  | M | 16 | 0.0054 (4e-04) |  |
| 30s | F | 22 | 0.0053 (6e-04) | U=374, p=0.56 |
|  | M | 31 | 0.0051 (3.7e-04) |  |
| 40s | F | 32 | 0.0050 (6e-04) | U=1057, p=0.35 |
|  | M | 59 | 0.0049 (6.8e-04) |  |
| 50s | F | 30 | 0.0043 (0.00134) | U=445, p=0.95 |
|  | M | 30 | 0.0042 (8.6e-04) |  |
| >60 | F | 4 | 0.0033 (3.9e-04) | U=9, p=0.61 |
|  | M | 6 | 0.0033 (3.2e-04) |  |
|  | | | | |
| **Supplementary Figure 4b (CSF Ab42)** | | | | |
| **Decade** | **Sex** | **N** | **Median (IQR)** | **Statistic (Mann-Whitney)** |
| 20s | F | 10 | 913 (654.8) | U=55, p=0.40 |
|  | M | 14 | 1062 (493) |  |
| 30s | F | 14 | 664 (834) | U=97, p=0.20 |
|  | M | 19 | 953 (730) |  |
| 40s | F | 40 | 558 (505.5) | U=986, p=0.79 |
|  | M | 51 | 590 (370.5) |  |
| 50s | F | 37 | 464 (237) | U=881, p=0.53 |
|  | M | 44 | 438 (246.8) |  |
| >60 | F | 4 | 446.5 (24.2) | U=0, p=0.13 |
|  | M | 2 | 640 (61) |  |
|  | | | | |
| **Supplementary Figure 4d (CSF Ab40)** | | | | |
| **Decade** | **Sex** | **N** | **Median (IQR)** | **Statistic (Mann-Whitney)** |
| 20s | F | 10 | 11464.5 (6255.2) | U=62, p=0.67 |
|  | M | 14 | 11381 (3039) |  |
| 30s | F | 14 | 9528.5 (8621.5) | U=110, p=0.42 |
|  | M | 19 | 12508 (6548) |  |
| 40s | F | 40 | 12181.5 (7960) | U=1058, p=0.76 |
|  | M | 51 | 10628 (5168) |  |
| 50s | F | 37 | 11189 (5404) | U=887.5, p=0.49 |
|  | M | 44 | 10260 (5762.5) |  |
| >60 | F | 4 | 11843 (1610.2) | U=0, p=0.13 |
|  | M | 2 | 16178.5 (2542.5) |  |
|  | | | | |
| **Supplementary Figure 4f (CSF t-tau)** | | | | |
| **Decade** | **Sex** | **N** | **Median (IQR)** | **Statistic (Mann-Whitney)** |
| 20s | F | 10 | 182.5 (83.5) | U=69, p=0.98 |
|  | M | 14 | 177 (75.8) |  |
| 30s | F | 14 | 191 (188.2) | U=88.5, p=0.11 |
|  | M | 19 | 318 (172) |  |
| 40s | F | 38 | 569.5 (560) | U=1142, p=0.15 |
|  | M | 51 | 375 (608) |  |
| 50s | F | 37 | 696 (665) | U=833, p=0.86 |
|  | M | 44 | 699 (505) |  |
| >60 | F | 4 | 961.5 (426) | U=2, p=0.53 |
|  | M | 2 | 1420.5 (457.5) |  |

# Supplementary Table 4. Detailed data and statistical values for Figure 3.

| **Figure 3a** | |  |  |  | | |  |
| --- | --- | --- | --- | --- | --- | --- | --- |
| **Age range** | **Sex** | ***APOE* Status** | **N** | **Frequency**  **Symptomatic cases** | | | **Statistic**  **(chi-squared test)** |
| 30-35 | F | E4 NC | 19 | 0 | | | na |
|  |  | E4 Carriers | 5 | 0 | | |  |
|  | M | E4 NC | 22 | 0 | | |  |
|  |  | E4 Carriers | 5 | 0 | | |  |
| 35-40 | F | E4 NC | 15 | 0 | | | na |
|  |  | E4 Carriers | 5 | 0 | | |  |
|  | M | E4 NC | 33 | 0 | | |  |
|  |  | E4 Carriers | 4 | 0 | | |  |
| 40-45 | F | E4 NC | 26 | 11.5 | | | X2 (3, N = 85) = 7.85,p=0.05 |
|  |  | E4 Carriers | 9 | 44.4 | | |  |
|  | M | E4 NC | 42 | 9.5 | | |  |
|  |  | E4 Carriers | 8 | 25 | | |  |
| 45-50 | F | E4 NC | 40 | 42.5 | | | X2 (3, N = 108) = 1.44,p=0.70 |
|  |  | E4 Carriers | 13 | 61.5 | | |  |
|  | M | E4 NC | 42 | 47.6 | | |  |
|  |  | E4 Carriers | 13 | 46.2 | | |  |
| 50-55 | F | E4 NC | 34 | 67.6 | | | X2 (3, N = 89) = 1.29,p=0.73 |
|  |  | E4 Carriers | 10 | 60 | | |  |
|  | M | E4 NC | 34 | 70.6 | | |  |
|  |  | E4 Carriers | 11 | 81.8 | | |  |
| 55-60 | F | E4 NC | 15 | 73.3 | | | X2 (3, N = 40) = 1.38,p=0.71 |
|  |  | E4 Carriers | 2 | 100 | | |  |
|  | M | E4 NC | 14 | 78.6 | | |  |
|  |  | E4 Carriers | 9 | 88.9 | | |  |
| >60 | F | E4 NC | 10 | 90 | | | na |
|  |  | E4 Carriers | 0 | - | | |  |
|  | M | E4 NC | 10 | 90 | | |  |
|  |  | E4 Carriers | 0 | - | | |  |
|  |  |  |  |  | |  | |
| **Figure 3c Data** | |  |  |  | |  | |
|  | **Sex** | ***APOE* Status** | **N** | **Mean (SD)** | |  | |
|  | F | E4 NC | 82 | 53.2 (5.5) | |  | |
|  |  | E4 Carriers | 27 | 50.5 (4.3) | |  | |
|  | M | E4 NC | 83 | 52.5 (5.4) | |  | |
|  |  | E4 Carriers | 30 | 52.2 (4.5) | |  | |
|  |  |  |  |  | |  | |
| **Figure 3c Between-group comparisons** | | | | | |  | |
|  | **Comparisons** | |  | | **Statistic (two-sample T-test)** | | |
|  | F.E4 NC vs F.E4 Carriers | | | | t(56.88) = 2.67, p = .010, d = 0.52 | | |
|  | F.E4 NC vs M.E4 NC | | | | t(162.89) = 0.79, p = .429, d = 0.12 | | |
|  | F.E4 NC vs M.E4 Carriers | | | | t(62.53) = 0.97, p = .337, d = 0.19 | | |
|  | F.E4 Carriers vs M.E4 NC | | | | t(55.75) = -2.02, p = .048, d = -0.40 | | |
|  | F.E4 Carriers vs M.E4 Carriers | | | | t(54.86) = -1.49, p = .141, d = -0.40 | | |
|  | M.E4 NC vs M.E4 Carriers | | | | t(61.34) = 0.31, p = .758, d = 0.06 | | |
|  |  |  |  |  | |  | |

*Abbreviations: F = Female; M = Male; E4 = APOE* ε4; NC = non-carrier.

# Supplementary Table 5: Characteristics of the study population used in the exploratory analyses assessing the interaction between sex and *APOE* ε4.

|  |  | **Female** | | **Male** | |  |
| --- | --- | --- | --- | --- | --- | --- |
|  |  | **E4 non-carriers**  **(n = 198)** | **E4 carriers**  **(n = 54)** | **E4 non-carriers**  **(n = 241)** | **E4 carriers**  **(n = 58)** | **p value** |
| **Age (years)** | | 45.3 [34.6;51.6] | 44.4 [35.5;49.3] | 43.1 [35.1;49.8] | 48.2 [38.0;51.6] | 0.230 |
| ***APOE* alleles, No.** | |  |  |  |  | NA |
|  | **ε2/ε2** | 0 (0.0%) | 0 (0.0%) | 2 (0.8%) | 0 (0.0%) |  |
|  | **ε2/ε3** | 26 (13.1%) | 0 (0.0%) | 28 (11.6%) | 0 (0.0%) |  |
|  | **ε2/ε4** | 0 (0.0%) | 6 (11.1%) | 0 (0.0%) | 2 (3.4%) |  |
|  | **ε3/ε3** | 172 (86.9%) | 0 (0.0%) | 211 (87.6%) | 0 (0.0%) |  |
|  | **ε3/ε4** | 0 (0.0%) | 44 (81.5%) | 0 (0.0%) | 54 (93.1%) |  |
|  | **ε4/ε4** | 0 (0.0%) | 4 (7.4%) | 0 (0.0%) | 2 (3.4%) |  |
| **Level of intellectual disability** | |  |  |  |  | 0.167 |
|  | **Mild** | 51 (26.4%) | 15 (27.8%) | 41 (17.2%) | 11 (19.3%) |  |
|  | **Moderate** | 95 (49.2%) | 31 (57.4%) | 136 (57.1%) | 30 (52.6%) |  |
|  | **Severe or profound** | 47 (24.4%) | 8 (14.8%) | 61 (25.6%) | 16 (28.1%) |  |
| **Diagnostic group** | |  |  |  |  | 0.179 |
|  | **Asymptomatic** | 123 (66.8%) | 32 (62.7%) | 162 (70.7%) | 32 (56.1%) |  |
|  | **Symptomatic AD** | 61 (33.2%) | 19 (37.3%) | 67 (29.3%) | 25 (43.9%) |  |
| **Medical conditions** | |  |  |  |  |  |
|  | **Hypothyroidism (n=395)** | 84 (59.2%) | 21 (53.8%) | 69 (39.9%) | 14 (34.1%) | 0.002 |
|  | **Epilepsy (n=328)** | 14 (11.4%) | 3 (9.7%) | 9 (6.5%) | 6 (16.7%) | 0.238 |
|  | **Sleep apnea (n=395)** | 11 (7.7%) | 4 (10.8%) | 25 (14.4%) | 8 (19.5%) | 0.117 |
|  | **Depression (n=422)** | 26 (17.3%) | 5 (12.2%) | 27 (14.5%) | 9 (20.0%) | 0.686 |
| **Cognition** | |  |  |  |  |  |
|  | **CAMCOG-DS scores (n=392)** | 73.0 [58.0;85.0] | 70.0 [51.5;82.0] | 70.0 [57.0;81.0] | 71.0 [60.8;83.2] | 0.528 |
|  | **mCRT Immediate recall (n=331)** | 35.0 [30.0;36.0] | 34.0 [21.0;36.0] | 35.0 [32.0;36.0] | 33.0 [26.8;35.8] | 0.173 |
|  | **mCRT Delayed recall (n=329)** | 12.0 [9.0;12.0] | 11.0 [6.2;12.0] | 12.0 [10.0;12.0] | 11.0 [8.2;12.0] | 0.317 |
| **Fluid biomarkers** | |  |  |  |  |  |
|  | **CSF Aβ1-42/1-40 (n=227)** | 0.1 [<0.1;0.1] | <0.1 [<0.1;0.1] | 0.1 [<0.1;0.1] | 0.1 [<0.1;0.1] | 0.182 |
|  | **CSF p-tau 181 (n=224)** | 64.2 [27.1;136.4] | 90.6 [53.8;155.3] | 54.7 [30.8;134.9] | 53.5 [26.5;119.6] | 0.455 |
|  | **CSF total tau (n=225)** | 471.5 [243.0;846.8] | 569.5 [330.8;1063.5] | 446.0 [262.0;853.0] | 475.5 [179.5;726.5] | 0.465 |
|  | **CSF NfL (n=149)** | 585.5 [301.9;930.0] | 589.3 [394.6;802.2] | 552.5 [357.4;1027.0] | 677.2 [313.0;835.0] | 0.964 |
|  | **Plasma p-tau 181 (n=505)** | 15.2 [9.0;25.1] | 14.9 [10.0;21.9] | 12.6 [8.6;22.3] | 14.9 [11.3;21.2] | 0.190 |
|  | **Plasma NfL (n=489)** | 12.5 [6.8;22.1] | 14.6 [9.0;20.7] | 10.6 [6.8;18.2] | 13.1 [7.8;22.4] | 0.184 |
| **Imaging biomarkers** | |  |  |  |  |  |
|  | **Centiloid amyloid PET (n=114)** | 14.7 [1.4;61.7] | 13.1 [7.2;40.1] | 11.5 [0.2;36.3] | 48.6 [8.9;80.2] | 0.134 |
|  | **FDG-PET SUVR (n=142)** | 1.3 [1.0;1.4] | 1.1 [0.8;1.1] | 1.3 [1.1;1.4] | 1.3 [1.0;1.4] | 0.535 |
|  | **Hippocampal volume (n=227)** | 5.6 [4.9;6.0] | 5.3 [4.6;5.7] | 6.2 [5.6;6.6] | 6.5 [5.0;6.9] | <0.001 |
|  | **Adj Hippocampal volume (n=227)** | <0.1 [<0.1;<0.1] | <0.1 [<0.1;<0.1] | <0.1 [<0.1;<0.1] | <0.1 [<0.1;<0.1] | 0.845 |
|  |  |  |  |  |  |  |

*Unless otherwise indicated, values represent n (%) or median [Quartile 1; Quartile 3]. All fluid biomarker concentration units, except for the CSF Aβ1-42/1-40 ratio, are pg/mL. P-values refer to analyses of chi-squared tests for categorical variables and Mann-Whitney tests for continuous variables. Abbreviations: Aß1-40 = amyloid β peptide 1–40. Aß1-42 = amyloid β peptide 1–42. CAMCOG-DS = Cambridge Cognitive Examination for Older Adults with Down syndrome. CSF = cerebrospinal fluid. FDG = ¹⁸Ffluorodeoxyglucose. mCRT = modified Cued Recall Test. NfL = neurofilament light chain. PET = positron emission tomography. p-tau181 = phosphorylated tau at threonine 181. SUVR = standardized uptake value ratio. TIV = Total intracranial volume.*

# Supplementary Table 6: Sample size for the exploratory analyses assessing the interaction between sex and *APOE* ε4 status.

|  |  | **Female** | | **Male** | |
| --- | --- | --- | --- | --- | --- |
|  |  | **E4 non-carriers**  **(n = 198)** | **E4 carriers**  **(n = 54)** | **E4 non-carriers**  **(n = 241)** | **E4 carriers**  **(n = 58)** |
| **Cognition** | |  |  |  |  |
|  | **CAMCOG-DS (n=392)** | N=141 | N=43 | N=168 | N=40 |
|  | **mCRT Immediate recall (n=331)** | N=124 | N=37 | N=140 | N=30 |
|  | **mCRT Delayed recall (n=329)** | N=125 | N=36 | N=138 | N=30 |
| **Fluid biomarkers** | |  |  |  |  |
|  | **CSF Aβ1-42/1-40 (n=227)** | N=82 | N=20 | N=101 | N=24 |
|  | **CSF p-tau 181 (n=224)** | N=80 | N=20 | N=100 | N=24 |
|  | **CSF NfL (n=149)** | N=55 | N=12 | N=65 | N=17 |
|  | **Plasma p-tau 181 (n=505)** | N=181 | N=50 | N=223 | N=51 |
|  | **Plasma NfL (n=489)** | N=177 | N=46 | N=216 | N=50 |
| **Imaging biomarkers** | |  |  |  |  |
|  | **Centiloid amyloid (n=114)** | N=37 | N=5 | N=55 | N=17 |
|  | **FDG-PET (n=142)** | N=57 | N=9 | N=60 | N=16 |
|  | **Hippocampal volume (n=227)** | N=73 | N=22 | N=106 | N=26 |

# Supplementary Fig. 1. Association between biological sex and cognitive performance analyzed by decades in adults with Down syndrome.


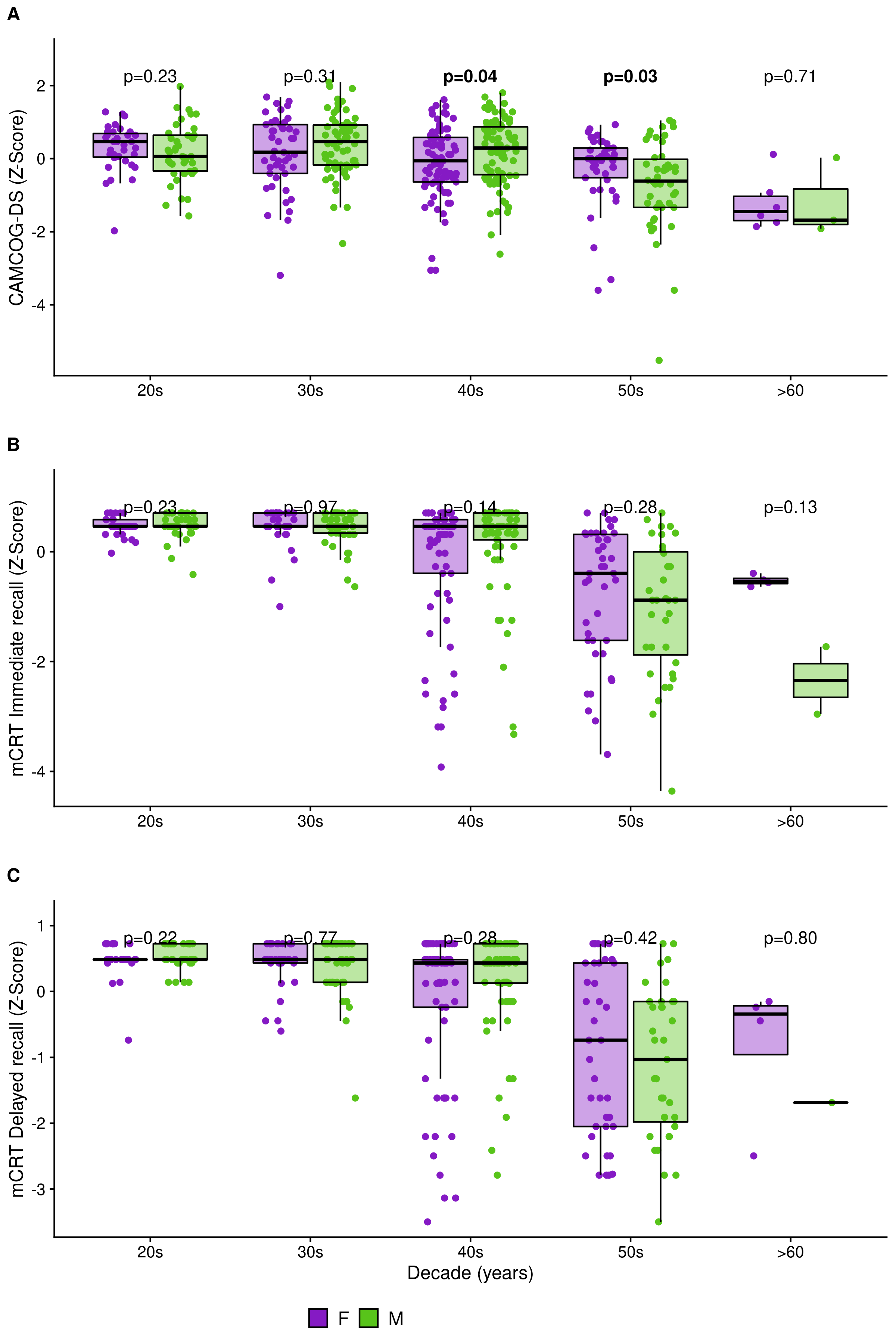


**Supplementary Fig. 1.** Boxplots showing the changes by decades in performances at the CAMCOG-DS (A), immediate (B), and delayed (C) recall at the mCRT test. *P* values refer to analyses of Mann-Whitney tests. The bold font indicates statistical significance (*P* < 0.05). Detailed data and statistical values are presented in Supplementary Table 3. CAMCOG-DS = Cambridge Cognitive Examination for Older Adults with Down Syndrome. mCRT = modified Cued Recall Test. F = female. M = male.

# Supplementary Fig. 2. Association between biological sex and cognitive performance in adults with Down syndrome with mild and moderate intellectual disability.


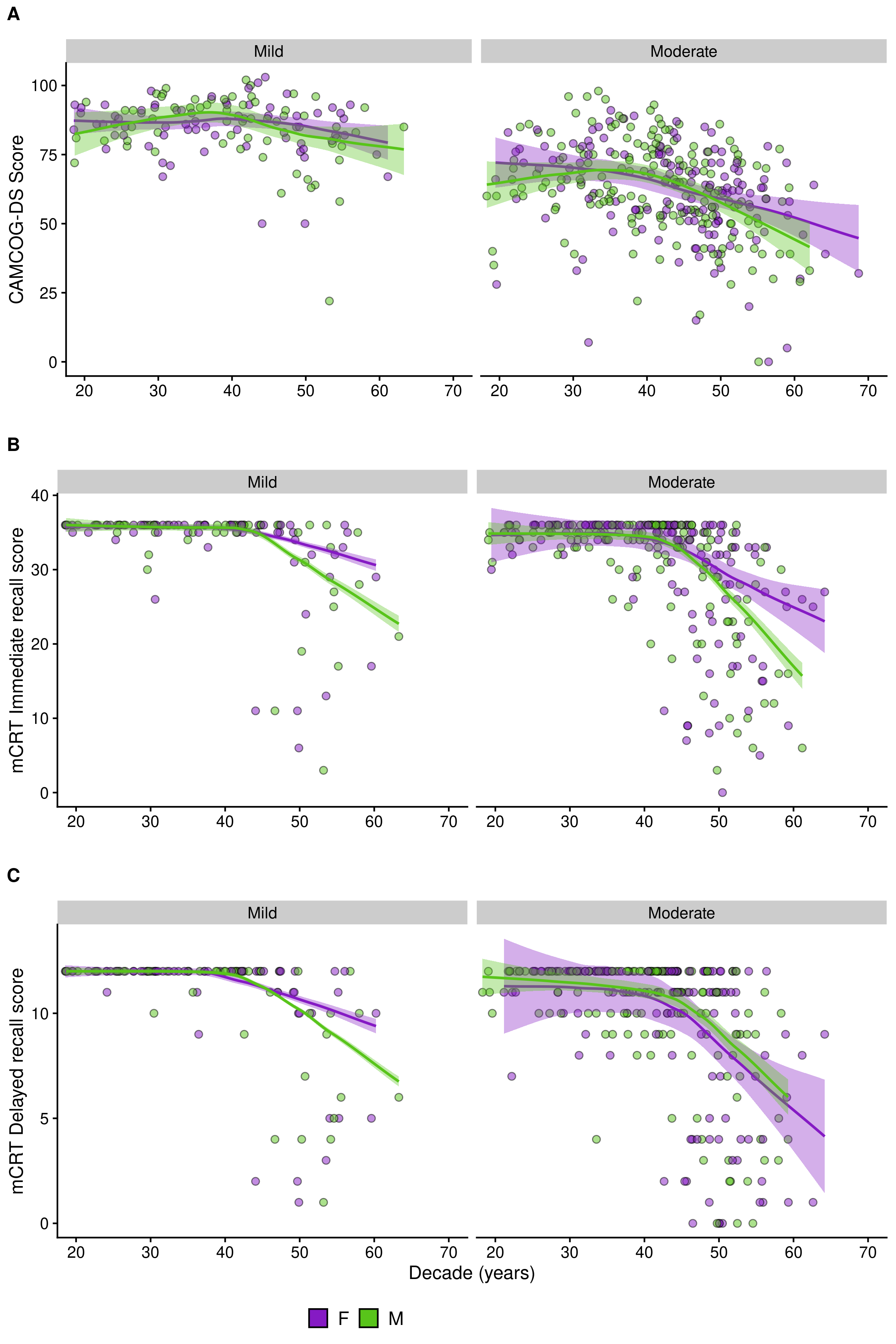


**Supplementary Fig. 2.** Scatterplots showing the age-related changes in performances at the CAMCOG-DS (A), immediate (B), and delayed (C) recall at the mCRT in individuals with mild and moderate intellectual disability, with bands representing the 95% confidence intervals. A significant difference between LOESS curves was defined as the age at which the curves diverged visually and the 95% confidence intervals did not overlap (p<0.05). CAMCOG-DS = Cambridge Cognitive Examination for Older Adults with Down Syndrome. mCRT = modified Cued Recall Test. F = female. M = male.

# Supplementary Fig. 3. Association between biological sex and Alzheimer’s disease biomarkers in adults with Down syndrome.


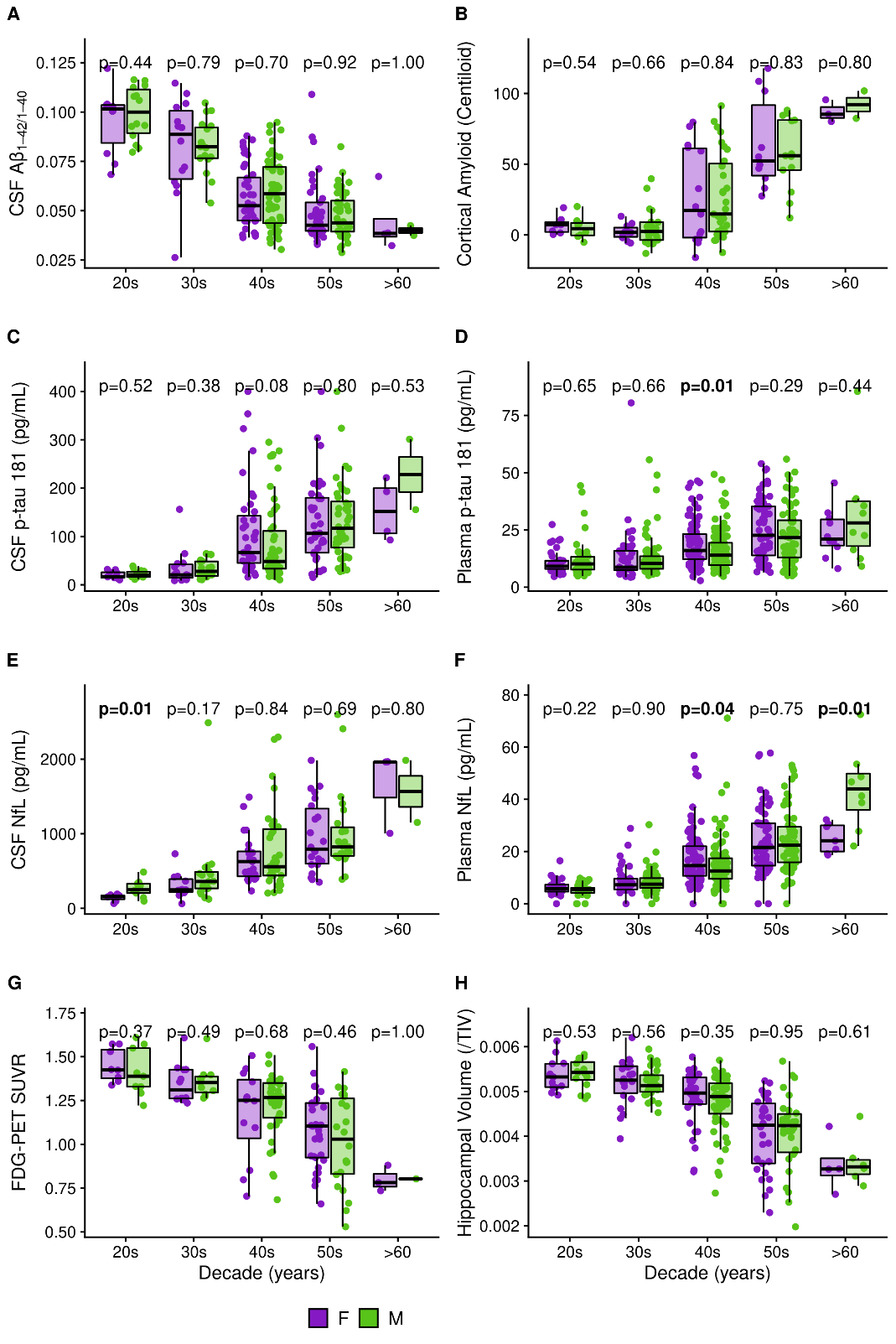


**Supplementary Fig. 3.** Boxplots showing the changes by decades in Alzheimer’s disease biomarkers. *P* values refer to analyses of Mann-Whitney. The bold font indicates statistical significance (*P* < 0.05). Detailed data and statistical values are presented in Supplementary Table 3. Aß_1-40_=amyloid β peptide 1–40. Aß_1-42_=amyloid β peptide 1–42. CSF=cerebrospinal fluid. FDG=¹⁸F-fluorodeoxyglucose. NfL=neurofilament light chain. SUVR=standardized uptake value ratio. TIV=Total intracranial volume. F = female. M = male.

# Supplementary Fig. 4. Association between biological sex and CSF levels of Aß_1-42_, Aß_1-40_, and total tau in adults with Down syndrome.


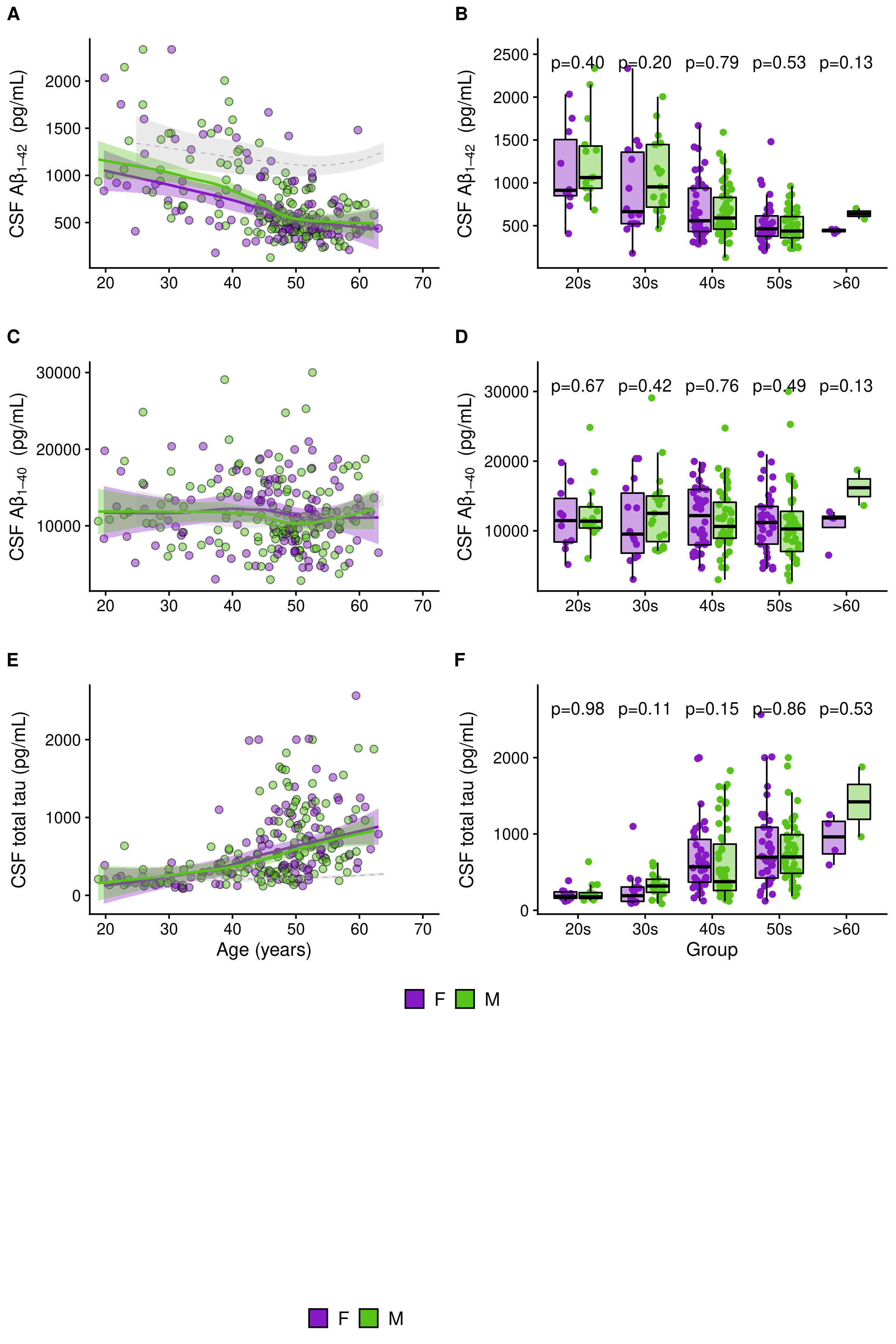


**Supplementary Fig. 4.** Scatterplots and boxplots showing the age-related changes of CSF Aß_1-42_ (panels A and B), CSF Aß_1-40_ (panels C and D, and CSF total tau (panels E and F) in adults with carriers with Down syndrome. Shading represents 95% confidence intervals. The grey lines represent the age-related changes in euploid individuals. A significant difference between LOESS curves was defined as the age at which the curves diverged visually and the 95% confidence intervals did not overlap (p<0.05). *P* values refer to analyses of Mann-Whitney. Detailed data and statistical values are presented in Supplementary Table 3. Aß_1-40_=amyloid β peptide 1–40. Aß_1-42_=amyloid β peptide 1–42. CSF=cerebrospinal fluid. F = female. M = male.
